# Single Phototrophic Bacterium-Mediated Iron Cycling in Aquatic Environments

**DOI:** 10.34133/research.0528

**Published:** 2024-11-18

**Authors:** Kai-Li Wang, Xin Ma, Dao-Bo Li, Yan-Ling Qi, Zheng-Shuang Hua, Tian Tian, Dong-Feng Liu, Di Min, Wen-Wei Li, Gui-Xiang Huang, Han-Qing Yu

**Affiliations:** ^1^ Department of Environmental Science and Engineering, University of Science and Technology of China, Hefei 230026, China.; ^2^ School of Life Sciences, University of Science and Technology of China, Hefei 230026, China.; ^3^ State Key Laboratory of Applied Microbiology Southern China, Institute of Microbiology, Guangdong Academy of Sciences, Guangzhou 510070, China.

## Abstract

Redox cycling of iron plays a pivotal role in both nutrient acquisition by living organisms and the geochemical cycling of elements in aquatic environments. In nature, iron cycling is mediated by microbial Fe(II)-oxidizers and Fe(III)-reducers or through the interplay of biotic and abiotic iron transformation processes. Here, we unveil a specific iron cycling process driven by one single phototrophic species, *Rhodobacter ferrooxidans* SW2. It exhibits the capability to reduce Fe(III) during bacterial cultivation. A *c*-type cytochrome is identified with Fe(III)-reducing activity, implying the linkage of Fe(III) reduction with the electron transport system. *R. ferrooxidans* SW2 can mediate iron redox transformation, depending on the availability of light and/or organic substrates. Iron cycling driven by anoxygenic photoferrotrophs is proposed to exist worldwide in modern and ancient environments. Our work not only enriches the theoretical basis of iron cycling in nature but also implies multiple roles of anoxygenic photoferrotrophs in iron transformation processes.

## Introduction

Iron is the most abundant transition metal on Earth, mainly existing in the forms of Fe(II) and Fe(III) species bound with hydroxyl, carbonate, sulfide, phosphate, and organic ligands [1–3]. Fe-organic matter complexes are widespread in aquatic environments. The redox potentials of iron species span a wide range from −314 mV to +770 mV in nature, bringing them high redox activity and varied solubility and bioavailability [4]. The iron cycling on modern Earth plays a pivotal role in the metabolism of aquatic organisms by supplying bioavailable iron as an essential nutrient [5,6]. On the other hand, the iron cycling also affects (bio)geochemical cycle of important elements (e.g., carbon, nitrogen, and sulfur) by directly involving in their redox transformations or element migration [7–9].

Fe(II)/Fe(III) cycling in natural environments is driven by both abiotic and biotic processes [10]. For example, Fe(II) can be oxidized by oxygen, manganese oxides, or reactive nitrogen species, while Fe(III) can be abiotically reduced in the presence of reduced natural organic matter or sulfur species, or by photolysis of Fe(III)-organic complexes. At the same time, microbial-driven Fe(III) reduction has been widely observed in various environments [11]. It is also well documented that Fe(II) is adopted as an electron donor by various bacteria and archaea, such as acidophilic *Acidithiobacillus* [12]; microaerophilic *Leptothrix*, *Mariprofundus*, *Sideroxydans*, and *Gallionella*; phototrophic *Rhodopseudomonas*, *Rhodobacter*, *Rhodovulum*, *Chlorobium*, and *Rhodomicrobium* [13]; and *Metallosphaera* species [14].

Microbial-mediated iron cycling, frequently observed in the environments exhibiting redox gradients, often requires close combinations between Fe(III)-reducers and Fe(II)-oxidizers [15]. Despite disparate optimal growth niches in terms of spatial distribution, these microorganisms were found to inhabit adjacent sites and drive microscale iron cycling in soil and sediments [16]. Moreover, the direction and strength of such an iron cycling are impacted by the organic carbon availability [17]. Considering the metabolic flexibility exhibited by certain Fe(II)-oxidizing bacteria with various terminal reductases connected to their electron transport chains, the mechanisms of iron cycling are more diverse in nature. For example, an acidophilic Fe(II)-oxidizer, *Acidithiobacillus ferrooxidans*, has been explicitly demonstrated to possess the capacity of Fe(III) reduction under acidic H_2_-incubation conditions in addition to its Fe(II)-oxidizing capability [18]. Likewise, a well-documented Fe(III)-reducer, *Shewanella putrefaciens* 200, was also found to oxidize Fe(II) coupled to nitrate reduction under circumneutral conditions [19]. However, since abiotic oxidation of Fe(II) by the intermediate products (e.g., nitric oxide and nitrite) of nitrate reduction exists, the biotic Fe(II)-oxidizing capacity of nitrate-reducing bacteria remains controversial [20,21]. Such a debate further raises a new doubt whether a single bacterium could drive iron cycling in circumneutral aquatic environments.

A recent work by Kato and Ohkuma [22] unveiled a neutrophilic chemolithoautotrophic Fe(II)-oxidizing *Rhodoferax* bacterium, strain MIZ03, that could oxidize Fe(II) as the sole electron donor under (micro)oxic conditions and reduce Fe(III) under anoxic conditions. This work lays a foundation for iron cycling by one organism in circumneutral aquatic environments. However, the enzymatic components governing such transformations of iron species remain enigmatic. Herein, we explored iron cycling processes mediated by the phototrophic Fe(II)-oxidizer *Rhodobacter ferrooxidans* SW2, a purple nonsulfur bacterium capable of using hydrogen and organic carbon as alternative electron donors in addition to Fe(II) for phototrophic growth [23]. Microbial reduction of both nitrilotriacetic acid (NTA)–complexed Fe(III) and poorly soluble hydrous ferric oxide [solid Fe(III)] and iron transformation processes by strain SW2 during continuous cultivation were investigated to verify the driving mechanism of iron cycling. A new *c*-type cytochrome (*c*-Cyt) with Fe(III) reduction activity was identified and suggested to dominate the Fe(III) reduction and contribute to the iron cycling by strain SW2. Coupled with phylogenetic analyses, our work suggests that single species of phototrophic Fe(II)-oxidizing bacteria are capable of driving iron redox cycling in aquatic environments and brings new understanding of the roles that anoxygenic photoferrotrophs played in iron transformation processes.

## Results

### Fe(III) reduction by *R. ferrooxidans* SW2

**Fig. 1.**
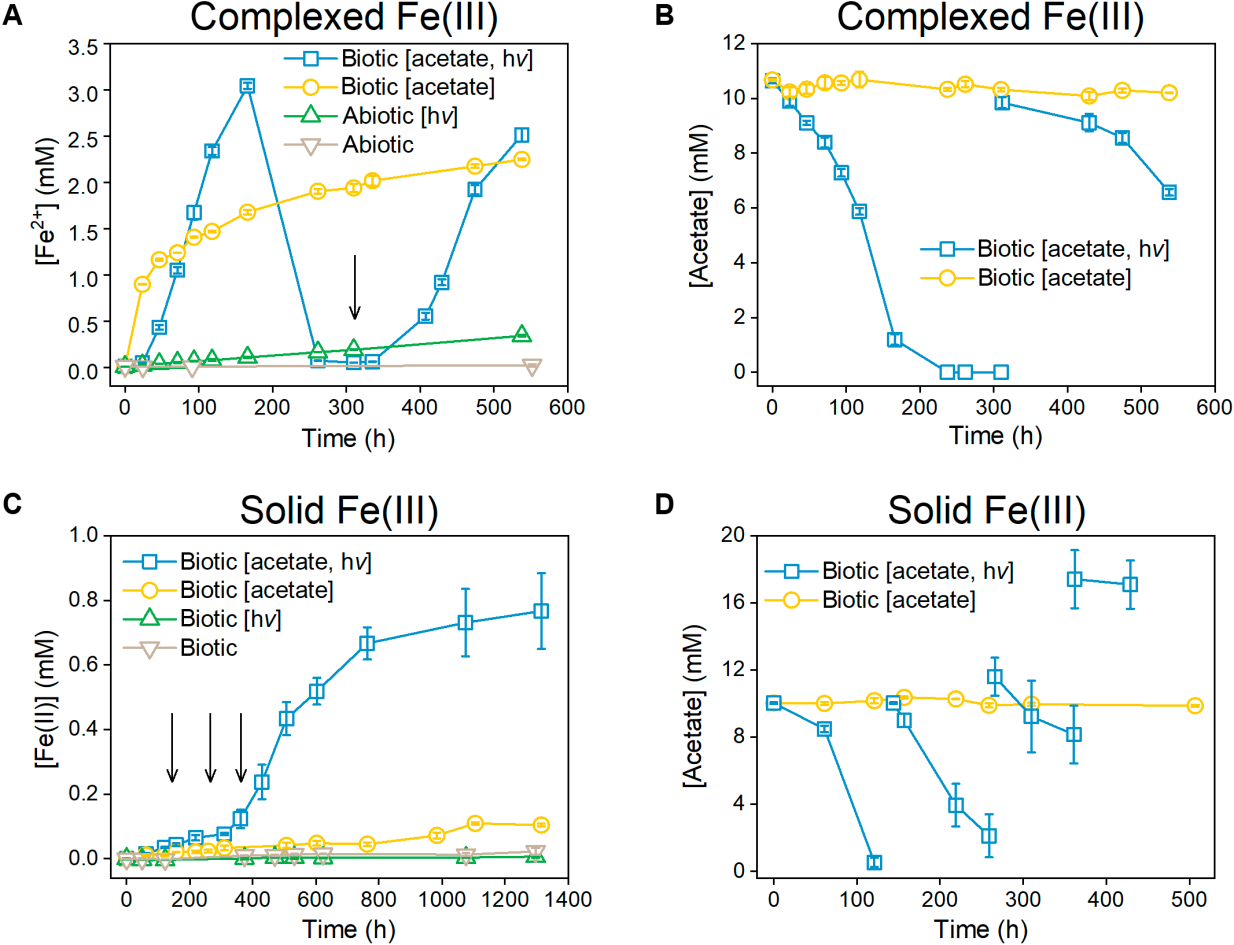
Fe(III) reduction and acetate consumption in the growth of *R. ferrooxidans* SW2. The changing profiles of (A) Fe(II) production and (B) acetate consumption in the complexed Fe(III) reduction cultures under illuminated and dark conditions. Cultures without strain SW2 incubation were adopted as abiotic controls. The changing profiles of (C) Fe(II) production and (D) acetate consumption in the solid Fe(III) reduction cultures under illuminated and dark conditions. Cultures without acetate amended were used as controls. Arrows [(A), 312 h; (C), 144, 266, and 362 h] indicate acetate replenishment (9.5 ± 0.2 mM) into the reduction cultures under illumination. OD_600_ = 0.3. Error bars indicate SD of triplicate tests.

During iron reduction process, electrons from organic or other compounds oxidized by microorganisms are transferred to Fe(III) species [24]. To demonstrate the Fe(III)-reducing activity of *R. ferrooxidans* SW2, both complexed Fe(III) and solid Fe(III) in the forms of NTA-Fe(III) and hydrous ferric oxide were used as the oxidants. During anaerobic phototrophic growth of *R. ferrooxidans* SW2 in the cultures containing acetate and Fe(III), the Fe(II) concentration rose continuously after a short lag phase (Fig. 1A and C). During Fe(III) reduction, simultaneous Fe(II) accumulation and acetate consumption were observed due to the photoheterotrophic growth of strain SW2 with acetate in the light, and specifically, up to 80% of Fe(III) was converted into Fe(II) (approximately 3.0 mM) until 166 h (Fig. 1A and B). Subsequently, the produced Fe(II) was rapidly consumed to a concentration below the detection limit after acetate became depleted. However, when acetate was replenished into the cultures, the Fe(III) reduction resumed immediately with a Fe(II) accumulation rate similar to that at the first Fe(III) reduction stage (0 to 166 h in Fig. 1A). Considering that each molecule of completely oxidized acetate provides 8 electrons, 4.0% and 9.4% of electrons from acetate oxidation were transferred to complexed Fe(III) at the first and second reduction (312 to 538 h) stages, respectively (Fig. S1). Notably, complexed Fe(III) reduction by *R. ferrooxidans* SW2 also occurred in the dark with no observable acetate consumption (Fig. 1A and B). The Fe(II) accumulation rate (approximately 1.7 mM at 166 h) in the dark was relatively lower compared to that under illumination. The abiotic and inactivated groups under illumination exhibited only slight Fe(II) generation (Fig. 1A and Fig. S2), mainly due to the light-driven intramolecular photoredox of NTA–complexed Fe(III) [25]. In addition, almost no Fe(II) could be determined in the abiotic and inactivated groups in the dark. Meanwhile, acetate addition did not trigger iron reduction in abiotic and inactivated groups under both illuminated and dark conditions either (Fig. S2). These results indicate that the observed Fe(III) reduction was predominantly driven by *R. ferrooxidans* SW2.

**Fig. 2.**
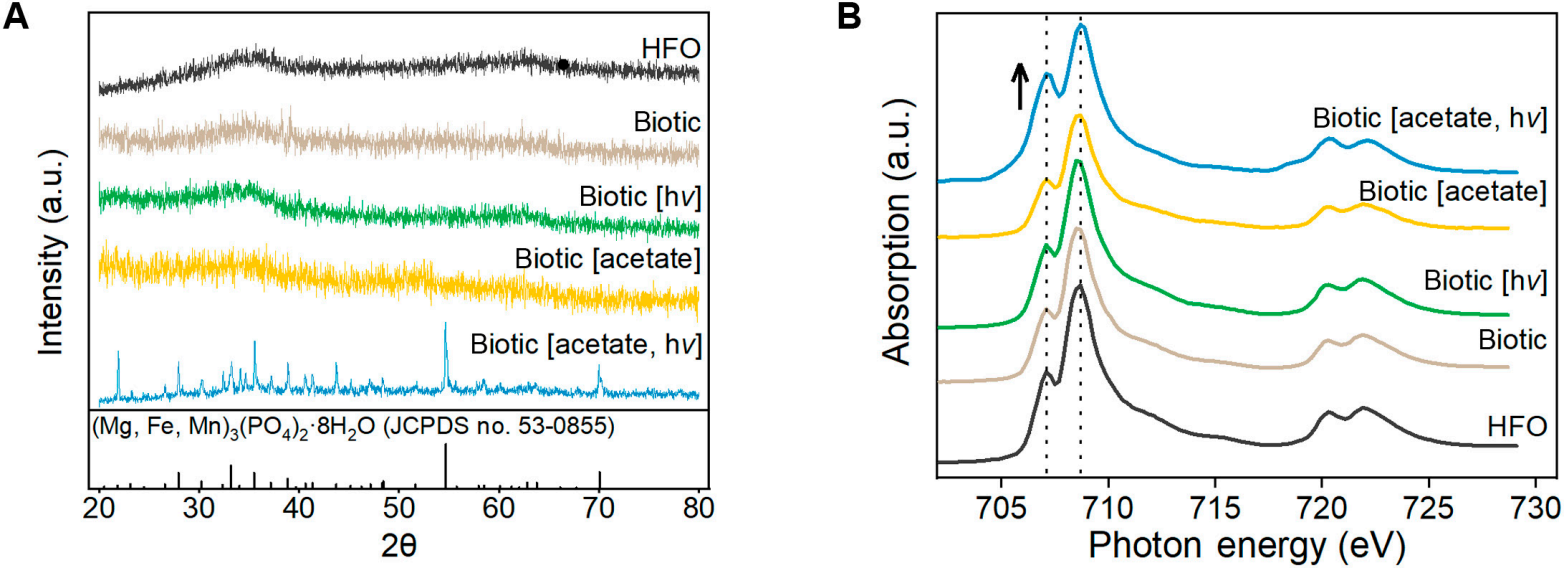
(A) X-ray diffraction and (B) Fe L_2,3_-edge XANES spectra characterization of the precipitates collected from the solid Fe(III) reduction cultures under various conditions. Samples collected from the acetate-free cultures were characterized as controls, and the chemically synthesized hydrous ferric oxide was characterized as a standard.

Compared to the complexed Fe(III), the solid Fe(III) was bio-reduced at a much lower rate. Accordingly, the Fe(II) finally accumulated up to 0.77 mM during the whole incubation period after repetitive acetate replenishments in the light (Fig. 1C and D). Only a modest amount of Fe(II) could be produced in the cultures incubated with acetate in the dark, which is consistent with a previous study [26]. The Fe(III) reduction did not occur without acetate supplementation, indicating that the solid Fe(III) reduction was microbial-driven reactions. The color of solid Fe(III) reduction cultures with acetate under illumination obviously changed from red to brown, accompanied by the formation of dark green precipitates (Fig. S3). Characterization of the deposited precipitates by x-ray powder diffraction (XRD) spectroscopy shows the presence of well-crystallized baricite [(Mg, Fe, Mn)_3_(PO_4_)_2_·8H_2_O, JCPDS card no. 53-0855] (Fig. 2A). In contrast, the precipitates collected from the dark cultures or the acetate-free illuminated culture all exhibited an amorphous morphology. The Fe L_3_-edge x-ray absorption near-edge structure (XANES) spectra (Fig. 2B) illustrate 2 distinct peaks at 707.1 and 708.5 eV for iron [27]. The relative peak intensity at 707.1 eV to that at 708.5 eV was enhanced in the acetate-fed *R. ferrooxidans* SW2 group under illumination rather than the others, revealing microbial reduction of solid Fe(III) to Fe(II).

**Fig. 3.**
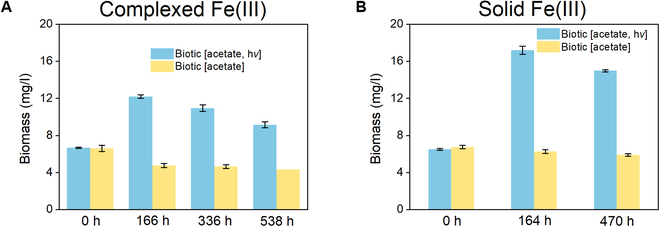
Biomass changes of *R. ferrooxidans* SW2 during the reduction of (A) the complexed Fe(III) and (B) the solid Fe(III) reduction with acetate under illuminated and dark conditions. OD_600_ = 0.3. Error bars indicate SD of triplicate tests.

The dynamics of protein content during reduction of complexed Fe(III) was monitored at given time intervals to evaluate the microbial growth. Data show that the protein content increased by 83% within 166 h under illumination (Fig. 3A), but slightly decreased at the subsequent stages of Fe(II) oxidation and restored Fe(III) reduction after acetate replenishment. The restrained biomass synthesis during the second stage of Fe(III) reduction was in accordance with the weaker acetate consumption (3.3 mM) than during the first stage (9.4 mM, Fig. 1B). Similar dynamics of protein content was observed for the solid Fe(III) reduction under illumination, with biomass first growing within the first 164 h and then slightly decaying (Fig. 3B). No protein content increase was observed in the reduction cultures under dark, indicating that the microbial growth of *R. ferrooxidans* SW2 was not supported by Fe(III) reduction under dark conditions.

### Identification of oxidoreductase for Fe(III) reduction

**Fig. 4.**
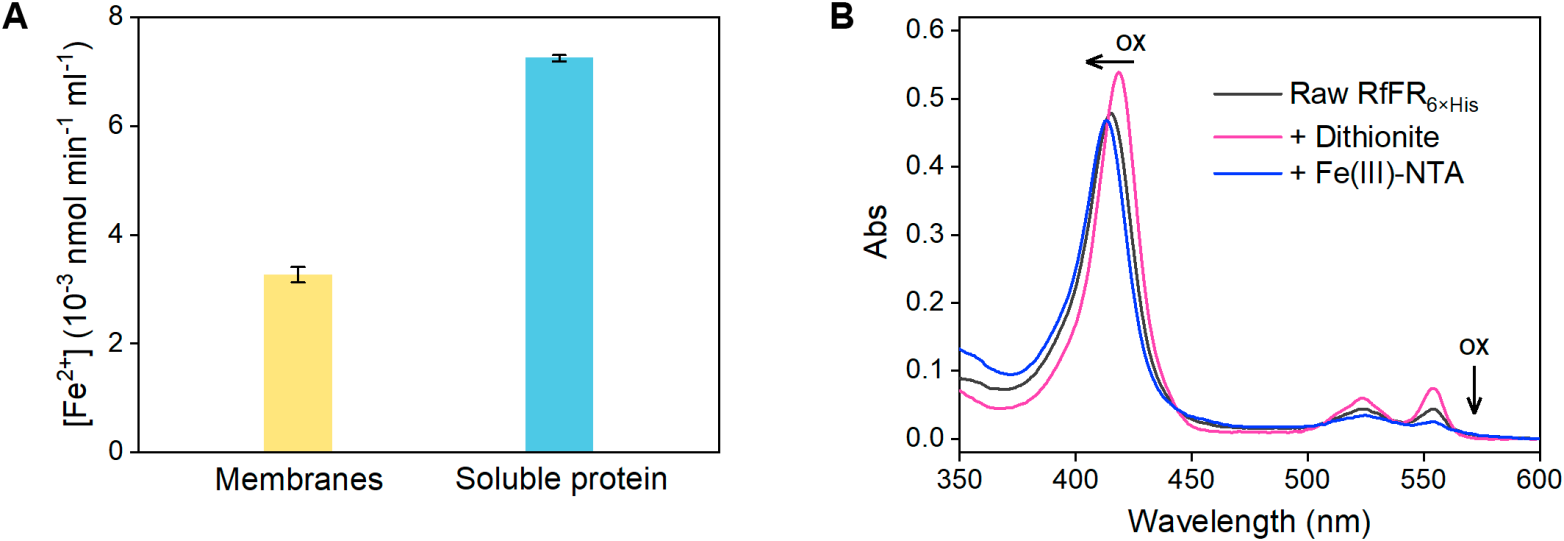
Identification of proteins responsible for Fe(III) reduction in *R. ferrooxidans* SW2. (A) Rates of Fe(III) reduction to Fe(II) [10^−3^ nmol of Fe(II) per milliliter sample per minute] catalyzed by insoluble and soluble fractions. Error bars indicate SD of triplicate tests. (B) UV–visible absorption spectra showing electron transfer from reduced RfFR to complexed Fe(III) (150 μM). Reduced RfFR was prepared by adding 20 μM dithionite. ox, oxidation.

After corroborating the Fe(III) reduction by *R. ferrooxidans* SW2, it is essential to further identify the enzymes responsible for such a Fe(III) reduction process. Many redox-active enzymes in phototrophic bacteria were located in the cytoplasmic membranes or periplasmic space, where photosynthetic and respiratory complexes of most purple bacteria are typically located [28]. Thus, the cells were disrupted and separated into membranes and soluble fractions. Interestingly, by using NADH [reduced form of nicotinamide adenine dinucleotide (oxidized form) (NAD^+^)] as an electron donor, both fractions retained complexed Fe(III)-reducing activities, but the soluble fraction exhibited a relatively higher one (Fig. 4A). Consequently, this fraction was treated using an anion exchange column for protein separation. Eluted subfractions with the dominant Fe(III) reduction activity showed 3 bands stained as *c*-Cyt in sodium dodecyl sulfate–polyacrylamide gel electrophoresis (SDS-PAGE) (Fig. S4). Through trypsin digestion and liquid chromatography–tandem mass spectrometry (LC-MS/MS) assay, *c*-Cyts encoded by genes *Rsw2DRAFT_0896*, *Rsw2DRAFT_0566*, and *Rsw2DRAFT_1533* were identified from these subfractions (Table S1). The fraction containing iron oxidoreductase FoxE that was previously assumed to catalyze Fe(III) reduction [29] unexpectedly exhibited a weak Fe(III)-reducing activity in our experiments (Fig. S4 and Table S1). Additionally, the proteins encoded by *Rsw2DRAFT_0896* and *Rsw2DRAFT_0566* were also identified in the supernatant of solid Fe(III) reduction cultures (Fig. S5 and Table S2), suggesting their possible involvement in the extracellular reduction of solid Fe(III) [30–32].

To further identify the specific enzymes, the genes *Rsw2DRAFT_0896*, *Rsw2DRAFT_0566*, and *Rsw2DRAFT_1533* were cloned for recombinant expression in the *c*-Cyt mutant (Δ*mtrCAB*-*omcA*-*mtrDFE*) of *Shewanella oneidensis* MR-1 (MR-1/Δ*mtr*), a strain suitable for synthesizing high levels of *c*-Cyts [33]. The expression of *c*-Cyts was confirmed by SDS-PAGE and LC-MS/MS analysis (Fig. S6 and Table S3). Ultraviolet (UV)–visible absorption spectra of these purified proteins were congruent with those of typical *c*-Cyts, with a Soret band between 408 and 418 nm and 2 Q bands between 523 and 554 nm (Fig. 4B and Fig. S7). To examine their Fe(III)-reducing activity, these proteins were respectively reduced with a minimal excess of dithionite and then mixed with complexed Fe(III). The protein encoded by *Rsw2DRAFT_0566* was reoxidized by complexed Fe(III), as evidenced by the UV–visible absorption spectra showing obvious changes in the peaks of Soret band and Q bands (Fig. 4B). In contrast, the proteins encoded by *Rsw2DRAFT_1533* and *Rsw2DRAFT_0896* could be hardly reoxidized even with excess amount of complexed Fe(III) (Fig. S7). Enzymatic assay for Fe(III) reduction by these proteins was performed to further evaluate their reduction activity toward Fe(III). The data show that the *c*-Cyt encoded by *Rsw2DRAFT_0566* exhibited a specific Fe(III) reduction activity that was 28 and 3 times higher than that of the proteins encoded by *Rsw2DRAFT_1533* and *Rsw2DRAFT_0896*, respectively (Table S4). These results clearly demonstrate that the protein encoded by *Rsw2DRAFT_0566*, here named as RfFR (*R. ferrooxidans* ferric reductase), provides *R. ferrooxidans* SW2 with dominant Fe(III)-reducing activity.

**Fig. 5.**
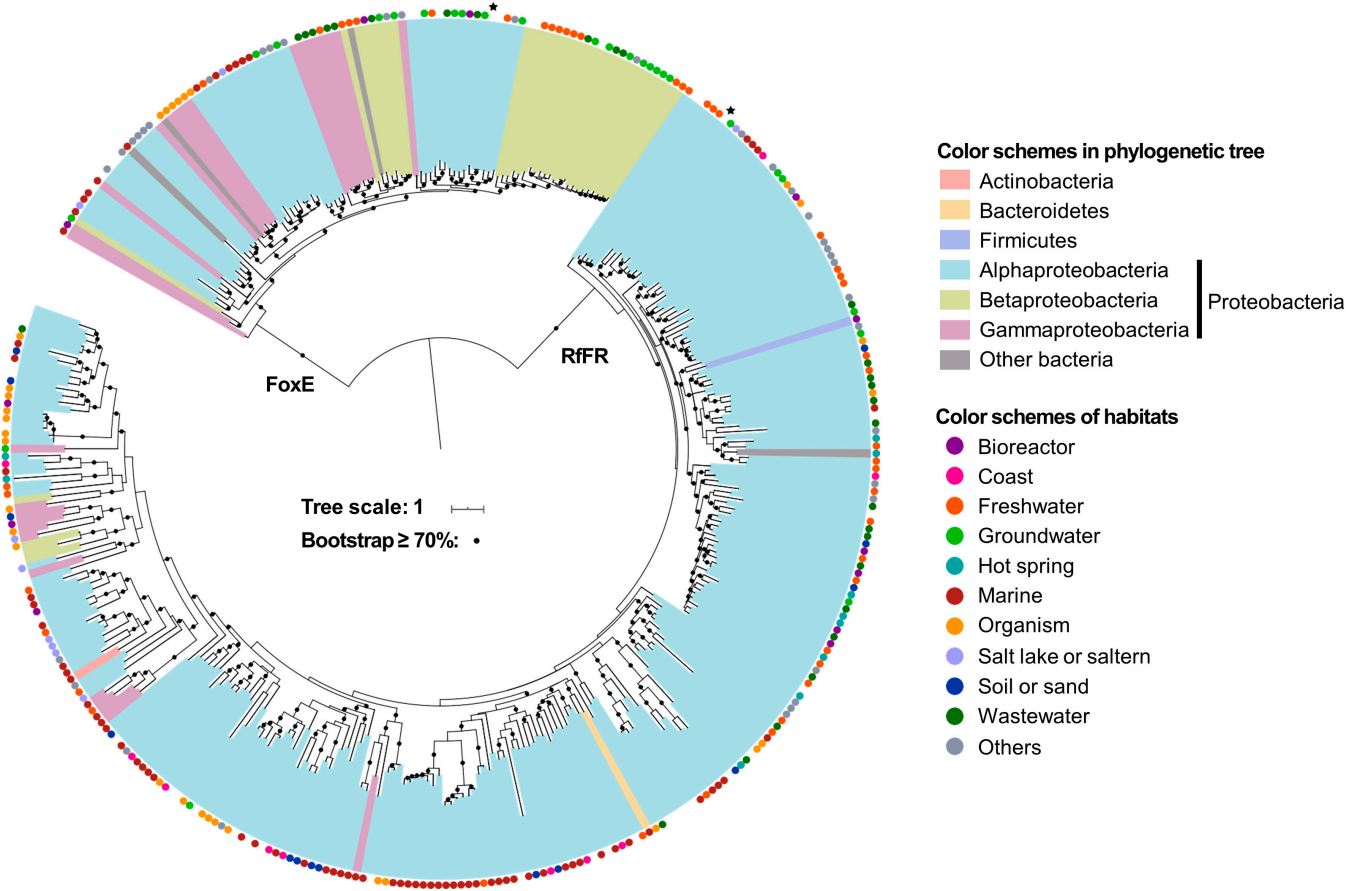
Phylogeny of FoxE and RfFR proteins. The phylogenetic tree was constructed using IQ-tree with WAG+F+R8 as the best model (see Methods). Bootstrap values >70% are shown in black circles. Black stars denote the *foxE* and *RfFR* genes from *R. ferrooxidans* SW2.

With the identified FoxE and RfFR proteins, we then reconstructed their phylogeny to reveal the universality of microbes harboring the *foxE* and/or *RfFR* genes that potentially drive iron cycling in the nature (Fig. 5). Most of *RfFR* genes are derived from *Alphaproteobacteria* that inhabit a broad range of ecosystems, such as freshwater, soil, and marine environments. The iron oxidase FoxE exhibits the same pattern as RfFR that only a narrow range of microbes possess this gene. Also, Alphaproteobacteria represents the biggest group and may bring other microbes with the ability to oxidize iron. In addition to *R. ferrooxidans* SW2, 4 extra isolates including *Alphaproteobacteria* strain CG_2015-68_30 (JAACUX01), *Rhodobacteraceae* strain ZodW_Metabat.71 (JAFGXY01), *Rhodobacterales* strain CG2_30_65_12 (MNZL01), and *Rhodobacteraceae* strain CG2_30_10_405 (MNZZ01) harbor both the *foxE* and *RfFR* genes. As a result, it is reasonable to infer that these microbes can also mediate iron cycling in natural environments.

### Iron redox cycling by *R. ferrooxidans* SW2

**Fig. 6.**
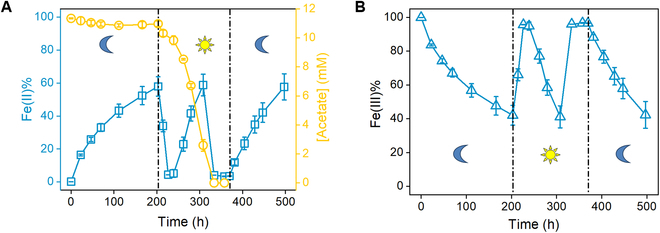
Switching between Fe(II) oxidation and Fe(III) reduction under light/dark conditions. Concentration dynamics of (A) Fe(II), acetate, and (B) Fe(III) in the Fe(III) reduction cultures [5 mM complexed Fe(III)] under light/dark change at 203 and 370 h with fresh cells. OD_600_ = 1.0. Error bars indicate SD of triplicate tests.

To explore the iron redox transformation under the regulation of light and organic substrates, *R. ferrooxidans* SW2 was inoculated at a high cell density (OD_600_ = 1.0) with complexed Fe(III) supplementation and subjected to alternating light–dark culturing. Concurrently, to validate the decrease in dissolved Fe(II) (Fig. 1A) that resulted from Fe(II) oxidation rather than precipitation, total iron analyses were performed under light/dark conditions (Fig. 6). A continuous Fe(II) accumulation without obvious acetate consumption was observed during dark cultivation (Fig. 6A), which was similar to those shown in Fig. 1A and B. This result indicates that advance intracellular electron donor(s) might be retained during its phototrophic growth before dark cultivation. Use of intracellular stored electrons or reducing equivalents for Fe(III) reduction was previously found in *Desulfotomaculum reducens* species [34]. Although such intracellular electron donors were not explicitly identified, the decrease in NADH/NAD^+^ ratio during the complexed Fe(III) reduction in the dark (Fig. S8) in this work indicated a concomitant turnover of NADH, a typical carrier for transferring intracellular electrons to other electron acceptors [35,36]. This result implied that *R. ferrooxidans* SW2 reserved intracellular electron donor(s) or sinks under illumination. When the Fe(III)-reducing culture in the dark was shifted to an illumination state after 203 h, the Fe(II) accumulation abruptly transitioned to a sharp Fe(II) consumption until 227 h. These results were presumably triggered by the deficiency of intracellular electron donor(s) for supporting Fe(III) reduction at the early stage of photoheterotrophic metabolism, although sufficient acetate was supplemented. After 227 h, the strains might generate adequate intracellular electron donor(s) from acetate metabolism under illumination, and thus, Fe(III) reduction was fully restored. The Fe(II) concentration continuously rose until acetate depletion, and then it declined over time. After acetate depletion, no further Fe(III) reduction occurred, although the culture time was lengthened (334 to 370 h), suggesting that acetate was essential for driving the microbial reduction of Fe(III) by *R. ferrooxidans* SW2 in the light. Subsequently, a remarkable Fe(II) rise was immediately triggered by shutting off the light. These results further demonstrated that the photoheterotrophic metabolism was indispensable for generating intracellular electron donor(s) [28] for subsequent Fe(III) reduction; otherwise, Fe(II) oxidation would prevail. Moreover, the opposite changes of total Fe(II) and Fe(III) (Fig. 6A and B) indicated a fluctuation of Fe(II) in the cultures, and a complete redox cycling of iron [i.e., from Fe(II) to Fe(III) and back to Fe(II) or vice versa] could be achieved by switching light and/or organic substrates.

## Discussion

Iron(III) oxy(hydr)oxides are prevalent in terrestrial and aquatic environments, and their formation and deposition on both modern and early Earth are associated with contributions from anoxygenic phototrophic Fe(II)-oxidizing bacteria [37,38]. In this work and several previous studies, *R. ferrooxidans* SW2 was found to oxidize Fe(II) in the cultures with little to no organic carbon under illumination, fitting well with typical photoautotrophic features [23,26]. Furthermore, in this work, we found that *R. ferrooxidans* SW2 could reduce Fe(III) both in the light and in the dark, while an upfront stage of photoheterotrophic metabolism with acetate as the carbon source and original electron donor is required. Rapid reversal between Fe(II) oxidation and Fe(III) reduction under illumination depends on the acetate availability in the cultures (Fig. 1A). Besides, Fe(III) reduction in the dark shifting to Fe(II) oxidation occurs in the initial hours of illumination (Fig. 6). These results indicate that redox cycling of iron can be driven by the switching of light and acetate. This is a special mechanism for iron cycling driven by single species of phototrophic bacteria, compared with the commonly known cooperation mechanism between differentiated Fe(II)-oxidizing and Fe(III)-reducing bacteria [15]. *R*. *ferrooxidans* SW2 reduces Fe(III) and induces a continuous accumulation of Fe(II) in the extracellular medium (Fig. 1A). Cells cultivated in both H_2_/CO_2_ (data not shown) and acetate cultures reduce Fe(III) in the dark, with kinetics of Fe(II) accumulation fitting well with the classical model of enzyme-catalyzed reactions (Fig. S9) [39]. These results indicate that electron donor(s) was stored intracellularly during phototrophic growth and then consumed as the reducing equivalents when Fe(III) was added into the cultures.

*c*-Cyts are widely involved in the electron transport processes of microorganisms. Multi-heme *c*-Cyts in several iron-respiring bacteria have been previously documented as Fe(III) reductases [40]. This work reports that the identified *c*-Cyt, RfFR, is a newly discovered enzyme catalyzing Fe(III) reduction. An alignment of peptide sequences shows that RfFR shared little sequence identity with membrane-anchored Fe(III) reductases (e.g., OmcA/MtrC and OmcS) [41,42] (Fig. S10). According to its protein sequence and spectral features, RfFR is a monoheme cytochrome *c* prime with a molecular weight of 14.0 kDa. Unlike those Fe(III) reductases containing multiple hemes for electron transfer across cellular membranes, RfFR is a mobile electron carrier located in the periplasmic space of *R*. *ferrooxidans* SW2 predicted by PSORTb. Besides, direct electron transfer from NADH to RfFR was also verified in the enzymatic assay. These results, along with the decrease in the NADH/NAD^+^ ratio during complexed Fe(III) reduction in the dark (Fig. S8), strongly suggest that RfFR was able to accept electrons from the respiratory complexes on the inner membrane to reduce Fe(III).

The special mechanism of iron cycling by *R. ferrooxidans* SW2 may exemplify a classic response to changes in the surrounding environment (e.g., light and organic substrates) in phototrophic Fe(II)-oxidizing bacteria. Although anoxic and iron-rich environments within the photic zone are rare on modern Earth, cryptic microbial iron cycling is very active, leading to high iron turnover in the low iron environments [43]. In addition, phototrophic Fe(II)-oxidizers are widely distributed on the land and ocean [13,44]. Among them, photoferrotrophic species with physiological and biochemical properties similar to *R. ferrooxidans* SW2, including *Chlorobium ferrooxidans*, *Rhodobacter capsulatus*, *Rhodopseudomonas palustris*, *Rhodomicrobium vannielii*, *Rhodovulum iodosum*, and *Rhodovulum robiginosum* isolated from aquatic environments [13], may also participate in such a complete iron cycling. In the case of iron reductases, multiple genes encoding *c*-Cyts of several phototrophic Fe(II)-oxidizing bacteria with potential Fe(III)-reducing activity have been predicted from their genomic information in the National Center for Biotechnology Information (NCBI) database (Table S5).

The above analyses suggest that local iron cycling mediated by photoferrotrophic species may be ubiquitous in aquatic environments, which extends far beyond previous expectations. In ancient ocean, anoxic Fe(II)-rich seawater could be oxidized by anoxygenic Fe(II)-oxidizing phototrophs mainly where light could reach. In modern aquatic environments, the increased oxygen levels make surface water oxygenated, leading to iron scavenging or precipitation from the water column. Residual dissolved iron is complexed with organic ligands to stay stabilized [5]. In the daytime, photoheterotrophic growth of anoxygenic phototrophs could be supported by light and organic substrates derived from nearby microbes or plants, accompanied by storage of intracellular electrons and reduction of Fe(III) species. When night falls, the prestored electrons could be consumed to further reduce Fe(III) species. As the sun rises again, the produced Fe(II) could be used again for their photoferrotrophic growth. The iron redox cycling mediated by these anoxygenic Fe(II)-oxidizing phototrophs depends on the availability of light and/or organics.

In summary, a single phototrophic species of *R. ferrooxidans* SW2 was discovered to drive the complete iron cycle in a circumneutral environment, which breaks the conventional perception that iron cycling requires either combinations between multiple microbial species or interactions between biotic and abiotic iron transformation. The direction of iron transformation in *R. ferrooxidans* SW2 is switchable, depending on the availability of light and/or organics. Fe(III) reduction occurs in the light and also in the dark if ample intracellular electron donors are stored from photoheterotrophic metabolism. The phylogenetic analysis infers that mainly Alphaproteobacteria have the potential to oxidize and reduce iron by one single organism, suggesting that these taxa presumably played a pivotal role in the iron cycle. These observations reveal a specific mechanism of iron cycling in natural aquatic environments and emphasize the necessity of reevaluating the role of phototrophic bacteria in iron (bio)geochemical cycling in both modern and ancient environments.

## Methods

### Bacterial strain and growth conditions

The strain *R. ferrooxidans* SW2 was gifted by A. Kappler at the University of Tuebingen, Germany. A mineral medium was prepared for microbial cultivation, which contained 0.1 g of CaCl_2_·2H_2_O, 0.3 g of NH_4_Cl, 0.5 g of MgSO_4_·7H_2_O, 0.5 g of KH_2_PO_4_, 1.0 ml of selenite–tungstate solution, 1.0 ml of trace element solution, and 1.0 ml of vitamin solution in 1 l of water. The selenite–tungstate solution contained 0.4 g l^−1^ NaOH, 0.008 g l^−1^ Na_2_WO_4_·2H_2_O, and 0.006 g l^−1^ Na_2_SeO_3_·5H_2_O. The trace element solution contained 25% HCl (10 ml), 0.03 g l^−1^ H_3_BO_3_, 0.19 g l^−1^ CoCl_2_·6H_2_O, 0.002 g l^−1^ CuCl_2_·2H_2_O, 0.036 g l^−1^ Na_2_MoO_4_·2H_2_O, 1.5 g l^−1^ FeCl_2_·4H_2_O, 0.1 g l^−1^ MnCl_2_·4H_2_O, 0.024 g l^−1^ NiCl_2_·6H_2_O, and 0.07 g l^−1^ ZnCl_2_. The vitamin solution contained 0.01 g l^−1^ D(+) biotin, 0.025 g l^−1^ Ca-D(+) pantothenate, 0.05 g l^−1^ thiaminium dihydrochloride, 0.05 g l^−1^ 4-aminobenzoic acid, 0.01 g l^−1^ nicotinic acid, 0.25 g l^−1^ pyridoxamine dihydrochloride, and 0.05 g l^−1^ cyanocobalamin. Oxygen was driven out of the medium by bubbling the solution with N_2_/CO_2_ (80:20, v/v), and the medium was immediately transferred into oxygen-free serum vials under N_2_/CO_2_ flushing treatments. Acetate and/or H_2_/CO_2_ (80:20, v/v) were supplied as electron donors. The serum vials were sealed and autoclaved. Afterward, the vitamin solution, selenite–tungstate solution, trace element solution, and bicarbonate (1.85 g/l) were sterilized via filtration through 0.22-μm sterilization filters and injected into the vials before the medium was used. The pH was adjusted to 7.0. The strain was cultivated at 23 °C under illumination with a 40-W tungsten lamp.

### Fe(III) reduction tests

*R. ferrooxidans* SW2 cells were harvested at stationary phase by centrifugation (6,000*g*, 8 min). For Fe(III) reduction, the cells were washed and added into fresh mineral medium at an OD_600_ of 0.3 or 1.0. Acetate (10 mM) or hydrogen was used as the external electron donor during Fe(III) reduction. Complexed Fe(III) (3.8 mM) or solid Fe(III) (0.96 g/l) was added as an electron acceptor in the reduction cultures. Acetate was replenished into the reduction cultures when it became almost completely depleted. Fe(III) reduction experiments with either sterile or inactivated bacteria were set as abiotic control groups. In complexed Fe(III) reduction cultures, samples were centrifuged to analyze dissolved Fe(II). To determine the total Fe(II) concentration in complexed and solid Fe(III) reduction cultures, samples were acidified with 6 M HCl to dissolve solid Fe(II). All experiments were conducted under anaerobic conditions.

NTA–complexed Fe(III) was prepared by completely dissolving 50 mmol NTA and 150 mmol NaOH in 100-ml boiled water and then adding 25 mmol Fe_2_(SO_4_)_3_ into it. Subsequently, the solution was degassed with N_2_ in a serum vial. The serum vial was sealed and shaken at room temperature for 2 h to obtain freshly made chelated Fe(III) (500 mM) solution. Hydrous ferric oxide was prepared according to a previous protocol with a slight modification [45]. NaOH was added slowly into Fe_2_(SO_4_)_3_ solution (0.4 M) with continuous stirring to adjust the pH to 7.0. The pellet was washed with distilled water for several times to remove salts. Then, it was freeze-dried and stored as a solid.

### Mineral characterizations

At the end of solid Fe(III) reduction test, minerals were collected by centrifugation (10,000*g*, 10 min), washed with oxygen-free water, and dried in an anaerobic glove box for subsequent characterizations. The XRD patterns of the minerals were obtained using a Philips X’ Pert PRO SUPER diffractometer (TTR-III, Rigaku Co., Japan) equipped with graphite monochromatized Cu Kα radiation (λ = 1.541874 Å). The Fe L_2,3_-edge XANES spectra were obtained at beamline BL12B in the National Synchrotron Radiation Laboratory in Hefei, China. XANES spectrum of hydrous ferric oxide was collected as a reference.

### Identification of Fe(III)-reducing components

*R. ferrooxidans* SW2 cells were harvested at stationary phase and lysed according to a previous protocol [46] with minor modification. Briefly, cells were washed with tris–HCl (10 mM, pH 8.0) and suspended in lysis buffer containing tris–HCl (10 mM, pH 8.0), lysozyme (1 mg ml^−1^), MgCl_2_ (1 mM), ribonuclease (8 μg ml^−1^), and deoxyribonuclease I (8 μg ml^−1^). Cell lysis was performed twice through a French pressure cell at 1,280 psi. Cell debris and unbroken cells were removed by centrifugation at 12,000*g* for 10 min. Supernatant was further centrifuged (115,000*g*, 1 h) to separate the soluble fraction from insoluble pellet (i.e., membranes). The soluble fraction was then separated to subfractions using a QHP anion exchange column (5 ml, GE Healthcare Co., USA). Main subfractions eluted from the column were collected for Fe(III) reduction assay and SDS-PAGE.

Fe(III) reduction activity of the subfractions and purified proteins was determined using the phenanthroline method. In a buffer containing 50 mM 3-(N-morpholino)propanesulfonic acid (Mops; pH 7.0) and 20 mM MgCl_2_, 1 mM NADH and an aliquot of subfraction were introduced as the electron donor and catalyzer, respectively. The mixture was incubated in an anaerobic glove box at 30 °C for 10 min. Then, 1 mM complexed Fe(III) and 3 mM 1, 10-phenanthroline of were dosed. The absorbance of the mixture at 510 nm was measured after 8 min.

For SDS-PAGE, the subfractions were individually mixed with the common sample buffer with the reducing agent being omitted. Heme staining was performed to the gel to identify *c*-Cyts. The stained slices were cut and digested with trypsin for protein identification in LC-MS/MS (Q Exactive Plus, Thermo Fisher Scientific Co., USA) analysis.

### Characterizations of Fe(III)-reducing components

Proteins encoded by the genes *Rsw2DRAFT_0566*, *Rsw2DRAFT_1533*, and *Rsw2DRAFT_0896* were expressed in MR-1/Δ*mtr*. The genes were cloned from the genome of *R. ferrooxidans* SW2 using primers listed in Table S6 and inserted into the vector pBAD202/D-TOPO [47]. In detail, oligos pBAD_For and pBAD_Rev were used to amplify the expression vector pBAD202/D-TOPO. The other oligos were used to amplify the responding gene fragments, and the polymerase chain reaction (PCR) products were ligated into the vector pBAD202/D-TOPO to afford pBAD-0566, pBAD-1533, and pBAD-0896. A 6×His tag was introduced to the C terminus to facilitate protein purification. The transformants were grown aerobically at 30 °C in a Terrific Broth medium containing 50 mg l^−1^ kanamycin. l-Arabinose (1 mM) was added into the medium at the middle of logarithmic phase to promote the expression of target proteins.

Target proteins were harvested and purified through cell lysis and affinity chromatography. The cells were pelleted from expression cultures by centrifugation (6,000*g*, 8 min), washed, and resuspended in 4-(2-hydroxyethyl)-1-piperazineethane-sulfonic acid (Hepes) buffer (20 mM Hepes, 100 mM NaCl, pH 7.8). Cell lysis was performed using sonication. Soluble proteins were collected from the lysate by centrifugation (12,000*g*, 10 min). The supernatant was collected and filtered through 0.45-μm membranes. The filtrate was then loaded onto a Ni-NTA Sepharose column (10 ml, Sigma-Aldrich Co., USA) preequilibrated with Hepes buffer, and washed with Hepes buffer containing 5 mM imidazole. Target proteins were eluted with Hepes buffer containing 40 mM imidazole, followed by redissolving into the Mops buffer by buffer change.

Fe(III) reduction by these purified *c*-Cyts was tested through in vitro assay. Dithionite was used to make reduced form of the purified *c*-Cyts. Complexed Fe(III) (50 to 500 μM) was mixed with reduced *c*-Cyts (OD_408_ = 0.3 to 0.5) in the Hepes buffer for 5 min at room temperature, and electron transfer was probed by the changes of UV–visible absorbance between 350 and 600 nm (UV-2450, Shimadzu Co., Japan).

### Phylogenetic analysis of FoxE and RfFR

Reference gene sequences of *foxE* and *RfFR* were collected and retrieved from both archaea and bacteria genomes available in NCBI-RefSeq database (downloaded on 2021 August 8). Protein coding sequences (CDSs) were predicted using Prodigal v2.6.3 [48] with the “-p single” option for all the downloaded complete/draft genomes (342,639 genomes). All identified CDSs were BLAST-searched against the FoxE and RfFR protein sequences retrieved from *R. ferrooxidans* SW2 using DIAMOND v0.8.22.84 [49] (*E* < 1 × 10^−5^). BLAST hits with identity >30% and coverage >60% of the subject sequence were retained for the later analysis. Functional annotation was conducted for RfFR gene sequences by comparing BLAST hits against the Kyoto Encyclopedia of Genes and Genomes (KEGG) [50], evolutionary genealogy of genes: nonsupervised orthologous groups (eggNOG) [51], and the Pfam protein families (Pfam) databases [52] using DIAMOND program (*E* < 1 × 10^−5^). Sequences without the identification of “cytochrome *c*” domain were removed. To avoid the redundancy, protein sequences from *foxE* and *RfFR* genes were clustered separately using CD-HIT v4.6 [53] with 95% sequence identity as the cutoff and only representatives were picked out for phylogeny reconstruction. Protein sequences from both genes were combined and aligned using MUSCLE v3.8.31 [54] by iterating 100 times. The poorly aligned regions were eliminated using TrimAl v1.4.rev22 [55] with the following parameters: -gt 0.05 -cons 50. The maximum likelihood phylogeny of FoxE and RfFR was inferred using IQ-tree v1.6.10 [56] with 1,000 ultrafast bootstrapping. The best model was determined by ModelFinder [57], which is well supported by Bayesian information criterion (BIC).

### Analytical methods

The concentration of Fe(II) was determined using the absorbance of Fe(II)-phenanthroline at 510 nm in acetic acid–ammonium acetate buffer. The concentration of acetate was determined using a gas chromatograph (GC 6890, Agilent Co., USA). Samples from Fe(III) reduction cultures were filtered. Aliquots from each filtered sample were acidified with 3% formic acid before loading into the column of the gas chromatograph. The intracellular NADH/NAD^+^ ratio was determined using a NAD^+^/NADH assay kit (Beyotime, China). The bacterial biomass in the Fe(III) reduction cultures was quantified by measuring the protein content. Briefly, the cells were suspended using 1 M NaOH and boiled at 100 °C for 5 min. The supernatant was analyzed using Micro BCA Protein Assay Kit (Sangon Biotech Co., China) following the manufacturer’s instructions.

## Data Availability

All data supporting the findings of this study are available in the paper and in the Supplementary Materials.
