# Supplementary material for: Single Phototrophic Bacterium-Mediated Iron Cycling in Aquatic Environments
Source: Research (Wash D C). 2024 Nov 18;7:0528. doi: 10.34133/research.0528 (PMC11570789; doi:10.34133/research.0528)
Supplement: Supplementary 1 — Figs. S1 to S10 Tables S1 to S6 [file research.0528.f1.zip › RESEARCH-D-24-01169-SM-Revised.DOCX]

**Supplementary Materials**

**Single Phototrophic Bacterium-Mediated Iron Cycling** **in Aquatic Environments**

Kai-Li Wang^1^, Xin Ma^2^, Dao-Bo Li^1,3^*, Yan-Ling Qi^1^, Zheng-Shuang Hua^1^, Tian Tian^1^, Dong-Feng Liu^1^, Di Min^1^, Wen-Wei Li^1^, Gui-Xiang Huang^1^, Han-Qing Yu^1^*

^1^Department of Environmental Science and Engineering; ^2^School of Life Sciences, University of Science and Technology of China, Hefei, 230026, China

^3^State Key Laboratory of Applied Microbiology Southern China, Institute of Microbiology, Guangdong Academy of Sciences, Guangzhou, 510070, China

**Supplementary Figures**


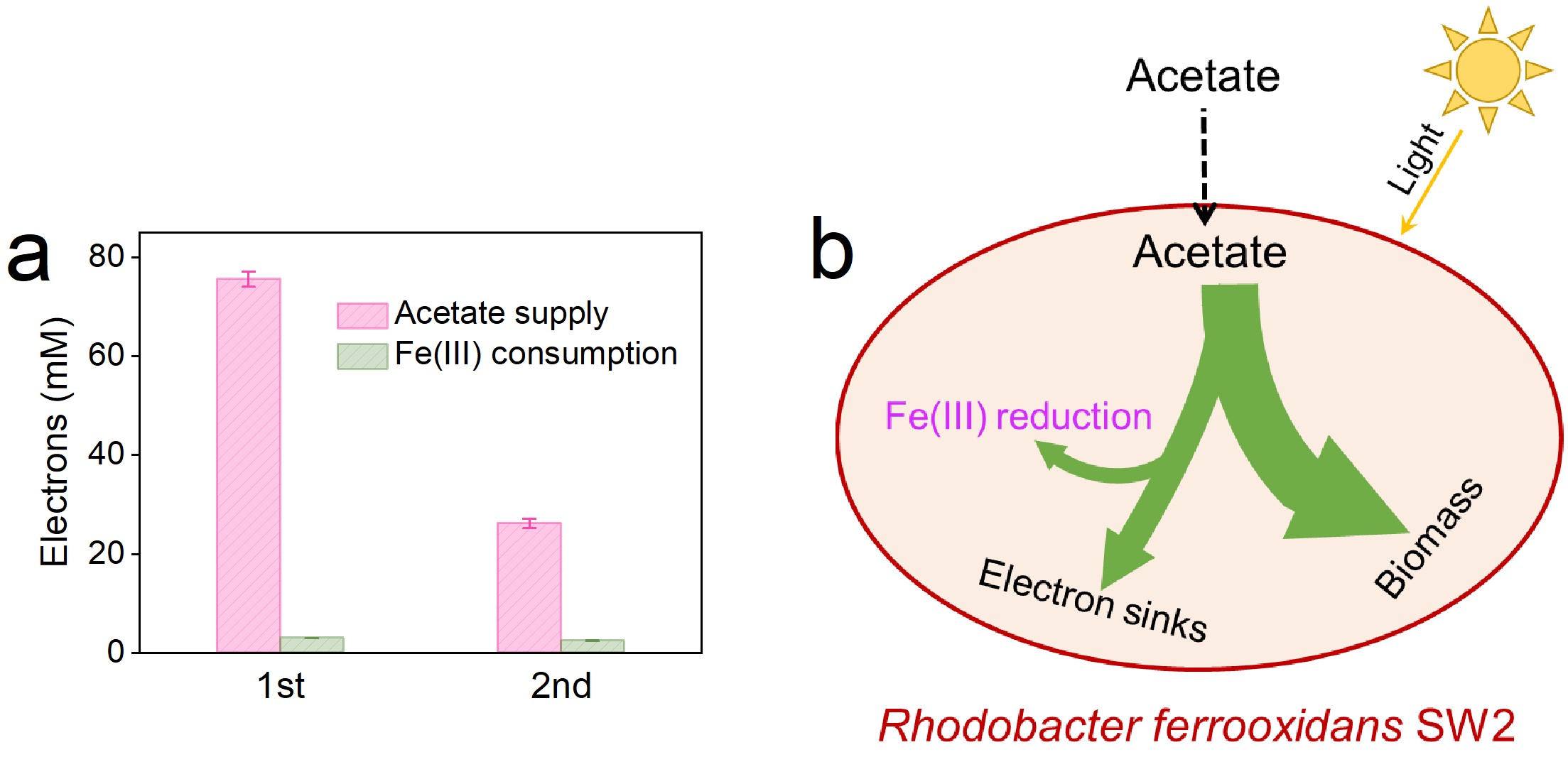


**Fig. S1.** Electron partitioning in *R. ferrooxidans* SW2 in acetate-supported Fe(III)-NTA reduction under illumination. (a) A comparison between electron release in complete acetate oxidation and electron spill to iron in Fe(III) reduction at the first (0-166 h) and second reduction stage (312-538 h) in Fig. 1a. (b) Electron partitioning diagram in *R. ferrooxidans* SW2. In acetate-supported Fe(III)-NTA reduction under illumination, most of acetate is used for biosynthesis while the others provide reducing power for intracellular electron sinks, only a portion of which are used for Fe(III) reduction.


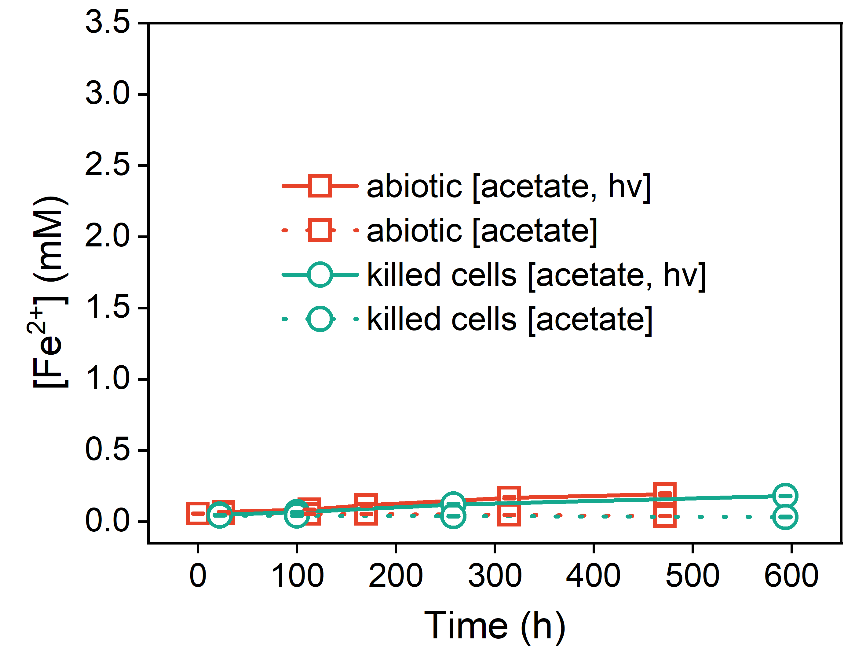


**Fig. S2.** Complexed Fe(III) reduction with acetate as electron donor in abiotic controls (inactivated or no cells) under illuminated and dark conditions.


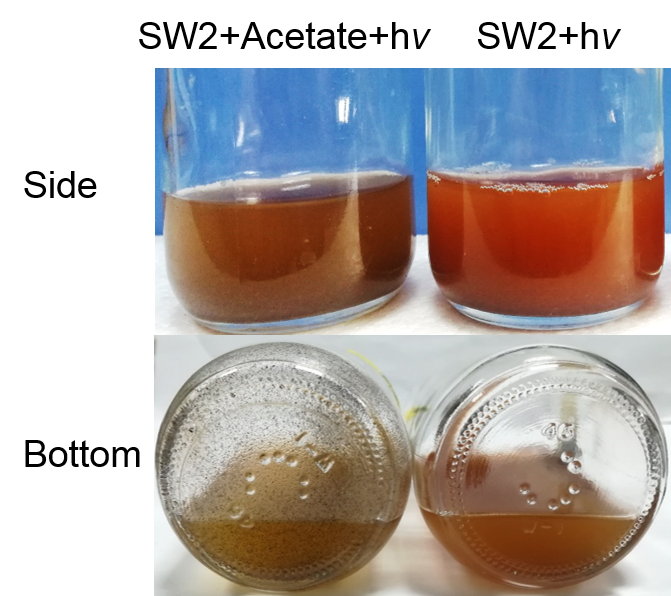


**Fig. S3.** Images of solid Fe(III) reduction cultures with (SW2+acetate+h*ν*) and without (SW2+h*ν*) acetate under illumination. OD_600_=0.3.


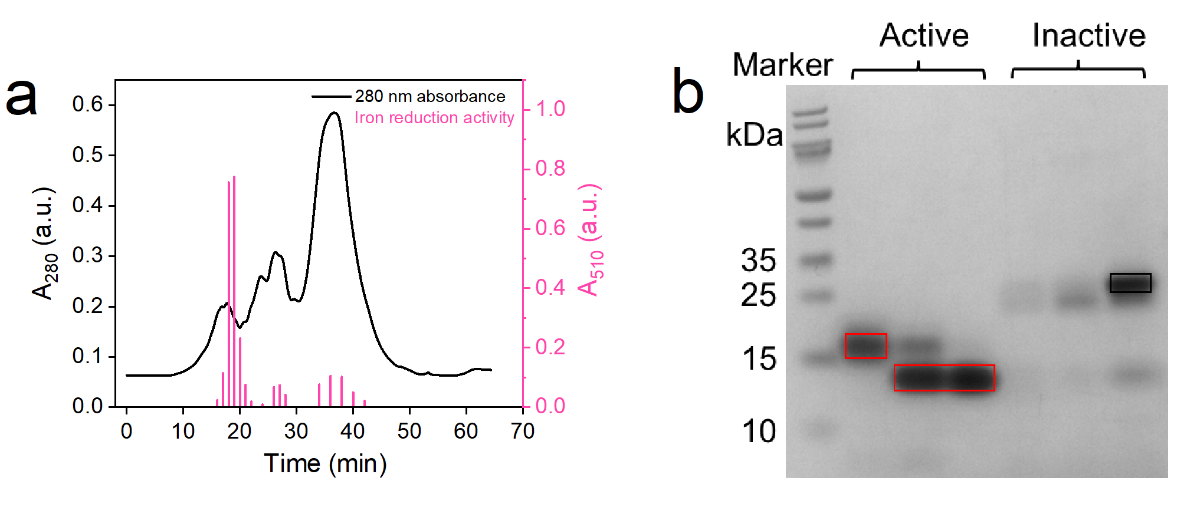


**Fig. S4.** Identification of the proteins with Fe(III)-reducing activity in the soluble fraction of *R. ferrooxidans* SW2 cells. (a) Separating the soluble fraction to the sub-fractions through an anion exchange column. Fe(III)-reducing activity was measured using the absorbance of Fe(II)-phenanthroline at 510 nm. (b) Separation of the proteins in the sub-fractions with different Fe(III)-reducing activity (active and inactive) using SDS-PAGE and heme staining. Gel slices containing *c*-Cyts in red and black boxes were cut for tryptic digestion and LC-MS/MS analysis.


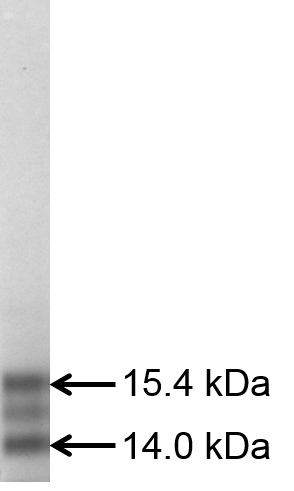


**Fig. S5.** SDS-PAGE and heme staining analysis of the supernatant from solid Fe(III) reduction cultures with acetate as electron donor under illumination. Stained bands were cut for tryptic digestion and LC-MS/MS analysis.


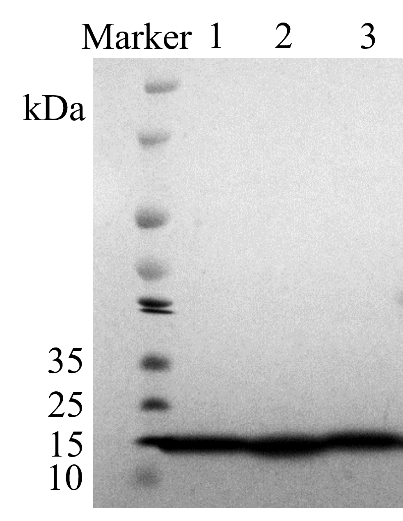


**Fig. S6.** SDS-PAGE analysis of purified *c*-Cyts encoded by genes of *Rsw2DRAFT_0566* (lane 1), *Rsw2DRAFT_1533* (lane 2), and *Rsw2DRAFT_0896* (lane 3).


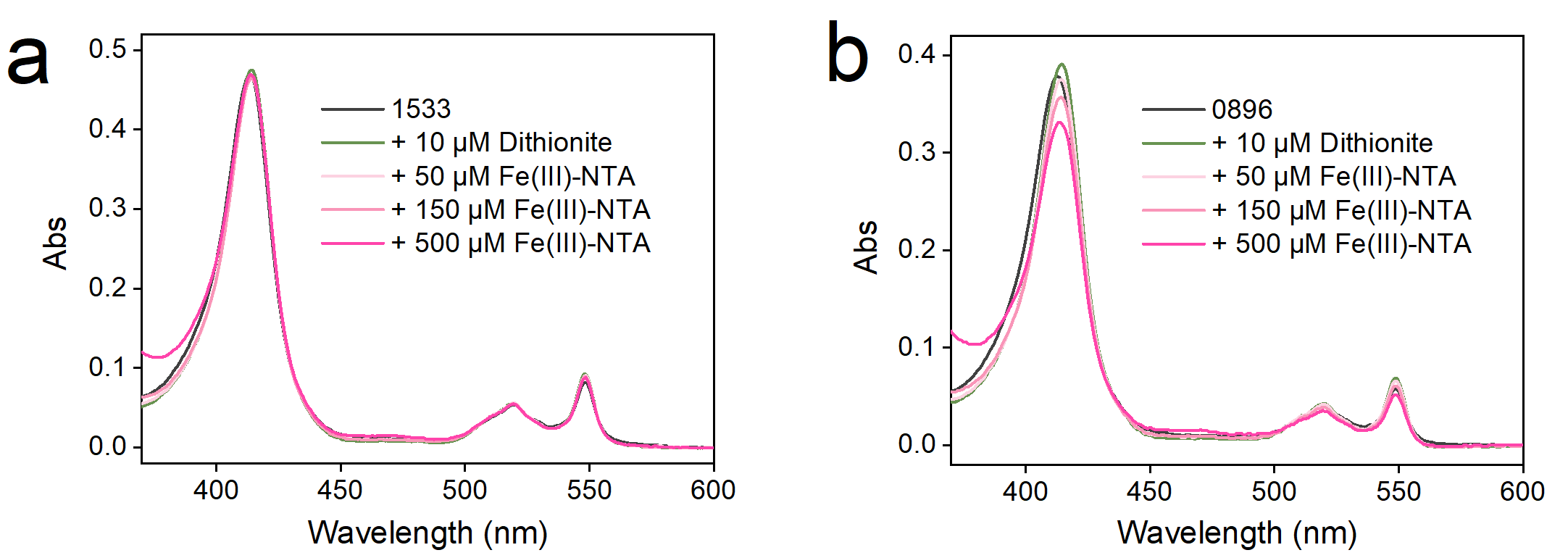


**Fig. S7.** Redox reactions of *c*-Cyts encoded by (a) Rsw2DRAFT_1533 and (b) Rsw2DRAFT_0896 with 10 μM dithionite and various concentrations of Fe(III)-NTA. The redox states of *c*-Cyts were monitored by UV-Visible absorption spectra.


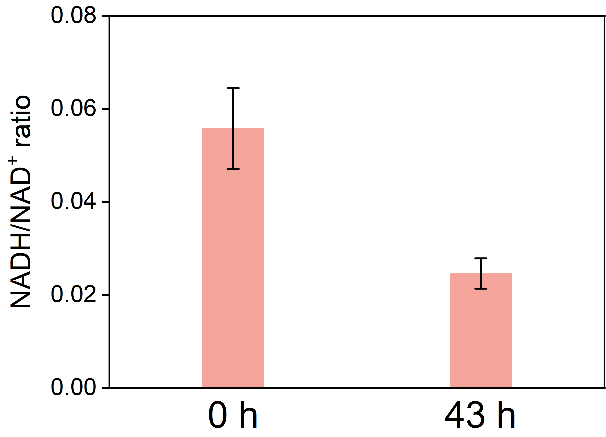


**Fig. S8.** Intracellular NADH/NAD^+^ ratio in the complexed Fe(III) reduction cultures in the dark.





**Fig. S9.** Kinetics of Fe(III)-NTA reduction by *R. ferrooxidans* SW2 in the dark in Fig. 1a. The fitting curve has the same grade as first-order kinetics.


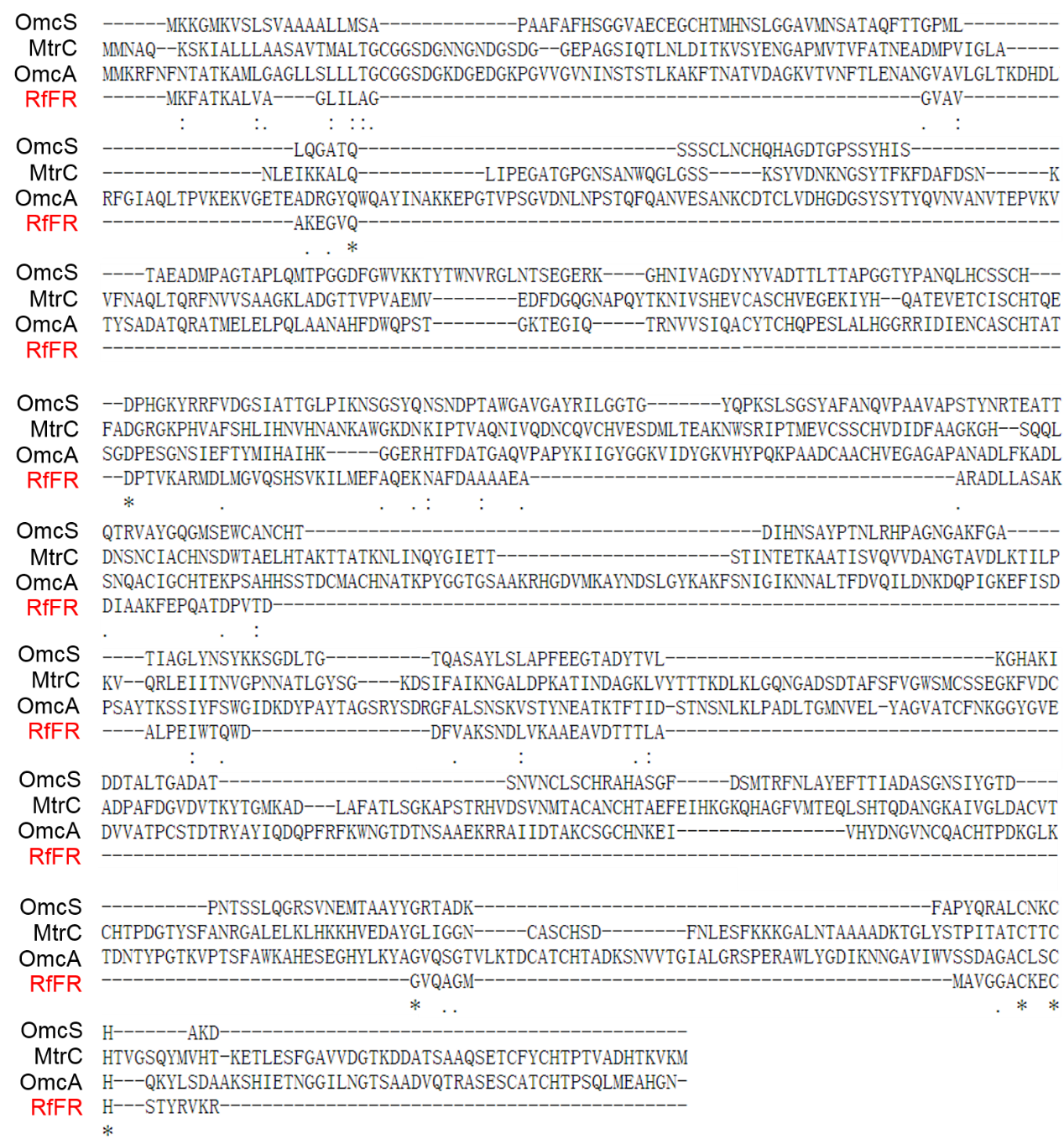


**Fig. S10.** Multiple alignments of RfFR with the membrane-anchored Fe(III) reductases (i.e., OmcS, MtrC and OmcA) from other species. Multiple sequence alignments of proteins were performed using MUSCLE. Semi-conserved (.), conserved (:) and identical (*) residues were displayed below the sequences.

**Supplementary Tables**

**Table S1.** The *c*-Cyts identified from the protein fractions with different Fe(III)-reducing activities (see Fig. S4b).

| fractions | accession name | description | gene name | protein mass (kDa) |
| --- | --- | --- | --- | --- |
| Active | C8RXN8_9RHOB | Cytochrome *c* prime | *Rsw2DRAFT_0566* | 14.0 |
|  | C8S0F5_9RHOB | Cytochrome *c* class I | *Rsw2DRAFT_1533* | 12.9 |
|  | C8RYL8_9RHOB | Cytochrome *c* class I | *Rsw2DRAFT_0896* | 15.4 |
| Inactive | A3DTD8_9RHOB | FoxE | *foxE* | 29.4 |

**Table S2.** The *c*-Cyts identified from the supernatant of solid Fe(III) reduction cultures.

| accession name | description | gene name | protein mass  (kDa) |
| --- | --- | --- | --- |
| C8RYL8_9RHOB | Cytochrome *c* class I | *Rsw2DRAFT_0896* | 15.4 |
| C8RXN8_9RHOB | Cytochrome *c* prime | *Rsw2DRAFT_0566* | 14.0 |
| C8RZ11_9RHOB | Cytochrome *c* prime | *Rsw2DRAFT_1039* | 14.9 |
| C8RYJ3_9RHOB | Cytochrome *c* prime | *Rsw2DRAFT_0871* | 14.3 |

**Table S3.** The *c*-Cyts identified from the gel slices shown in Fig. S6.

| lanes | accession name | description | gene name | protein mass (kDa) |
| --- | --- | --- | --- | --- |
| 1 | C8RXN8_9RHOB | Cytochrome *c* prime | *Rsw2DRAFT_0566* | 14.0 |
| 2 | C8S0F5_9RHOB | Cytochrome *c* class I | *Rsw2DRAFT_1533* | 12.9 |
| 3 | C8RYL8_9RHOB | Cytochrome *c* class I | *Rsw2DRAFT_0896* | 15.4 |

**Table S4.** Specific iron reduction activity of purified *c*-Cyts.

| purification  procedure | encoding gene | total protein  (μg) | iron reduction activity  (nmol Fe(II) formed min^−1^) | specific activity  (nmol Fe(II) formed mg  protein^−1^ min^−1^) |
| --- | --- | --- | --- | --- |
| crude extract | *Rsw2DRAFT_0566* | 23.3 | 9.06 | 388.3 |
|  | *Rsw2DRAFT_1533* | 23.0 | 6.76 | 293.4 |
|  | *Rsw2DRAFT_0896* | 26.6 | 9.33 | 350.4 |
| Ni-NTA Sepharose column | *Rsw2DRAFT_0566* | 10.0 | 0.47 | 46.9 |
|  | *Rsw2DRAFT_1533* | 18.2 | 0.03 | 1.7 |
|  | *Rsw2DRAFT_0896* | 15.3 | 0.26 | 16.9 |

**Table S5.** Possible *c*-Cyts in several phototrophic Fe(II)-oxidizing bacteria predicted from their genomic information in NCBI.

| No. | strains | counting of *c*-Cyts | accession | localization^*^ |
| --- | --- | --- | --- | --- |
| 1 | *Chlorobium ferrooxidans* | 8 | WP_006365223.1 | - |
|  |  |  | WP_006365497.1 | CM |
|  |  |  | WP_006367224.1 | - |
|  |  |  | WP_006367389.1 | - |
|  |  |  | WP_006367150.1 | - |
|  |  |  | WP_006367149.1 | PS |
|  |  |  | WP_006367148.1 | PS |
|  |  |  | WP_006366394.1 | - |
| 2 | *Chlorobium phaeoferrooxidans* | 7 | ABL66452.1 | PS |
|  |  |  | ABL66451.1 | PS |
|  |  |  | ABL66116.1 | PS |
|  |  |  | ABL65302.1 | CM |
|  |  |  | ABL64616.1 | CM |
|  |  |  | ABL64564.1 | - |
|  |  |  | ABL64509.1 | CM |
| 3 | *Chlorobium sp. strain N1* | 7 | TCD48959.1 | - |
|  |  |  | TCD48798.1 | - |
|  |  |  | TCD48755.1 | CM |
|  |  |  | TCD48574.1 | - |
|  |  |  | TCD48048.1 | - |
|  |  |  | TCD47735.1 | PS |
|  |  |  | TCD47734.1 | - |
| 4 | *Rhodobacter capsulatus* | 18 | WP_136905964.1 | PS |
|  |  |  | WP_081348877.1 | - |
|  |  |  | WP_169583102.1 | PS |
|  |  |  | WP_157851872.1 | - |
|  |  |  | WP_212635373.1 | CM |
|  |  |  | WP_152970447.1 | CM |
|  |  |  | WP_136906067.1 | CM |
|  |  |  | WP_055208593.1 | PS |
|  |  |  | WP_023913195.1 | PS |
|  |  |  | WP_136906066.1 | PS |
|  |  |  | WP_136904505.1 | PS |
|  |  |  | WP_136906054.1 | CM |
|  |  |  | WP_136905968.1 | - |
|  |  |  | WP_136905099.1 | - |
|  |  |  | WP_031321502.1 | CM |
|  |  |  | WP_136906992.1 | PS |
|  |  |  | WP_136905190.1 | - |
|  |  |  | WP_136905217.1 | CM |
| 5 | *Rhodopseudomonas palustris* | 2 | QQM05533.1 | PS |
|  |  |  | QQM05245.1 | CM |
| 6 | *Rhodomicrobium vannielii* | 3 | WP_201719580.1 | - |
|  |  |  | WP_201719122.1 | CM |
|  |  |  | WP_201718712.1 | PS |
| 7 | *Rhodovulum robiginosum* | 21 | RSK37847.1 | PS |
|  |  |  | RSK37809.1 | - |
|  |  |  | RSK36482.1 | - |
|  |  |  | RSK33655.1 | - |
|  |  |  | RSK33478.1 | CM |
|  |  |  | RSK33476.1 | CM |
|  |  |  | RSK33471.1 | - |
|  |  |  | RSK33440.1 | PS |
|  |  |  | RSK32960.1 | CM |
|  |  |  | RSK32942.1 | PS |
|  |  |  | RSK32844.1 | CM |
|  |  |  | RSK32787.1 | - |
|  |  |  | RSK32311.1 | - |
|  |  |  | RSK32309.1 | - |
|  |  |  | RSK32266.1 | PS |
|  |  |  | RSK32073.1 | CM |
|  |  |  | RSK31519.1 | CM |
|  |  |  | RSK31455.1 | - |
|  |  |  | RSK31454.1 | - |
|  |  |  | RSK30668.1 | - |
|  |  |  | ACN69110.1 | - |

^*^Predicted with PSORTb version 3.0.3.

CM: cytoplasmic membrane.

PS: Periplasmic space

**Table S6.** Primers used in this work.

| primers | sequences (5’-3’) | primer length | amplicon size |
| --- | --- | --- | --- |
| pBAD_For | CATCATCACCATCACCATTG | 20 | 3995 |
| pBAD_Rev | GGGTATGTATATCTCCTTCTTAAAG | 25 |  |
| 0566_For | GGAGATATACATACCCATGAAATTTGCCACC | 31 | 463 |
| 0566_Rev | GTGATGATGGCGTTTGACGCG | 21 |  |
| 1533_For | GGAGATATACATACCCATGAAAATCAGTCTC | 31 | 439 |
| 1533_Rev | GTGATGATGGTTGGTGGCGAC | 21 |  |
| 0896_For | AGAAGGAGATATACATACCCATGAAATTCACCCTCTCTGCCGCCGCC | 47 | 535 |
| 0896_Rev | CAATGGTGATGGTGATGATGGTTGCTGGGTGCCGGGGCGG | 40 |  |
