# Supplementary figures and images for: Single Phototrophic Bacterium-Mediated Iron Cycling in Aquatic Environments
Source: Research (Wash D C). 2024 Nov 18;7:0528. doi: 10.34133/research.0528 (PMC11570789; doi:10.34133/research.0528)

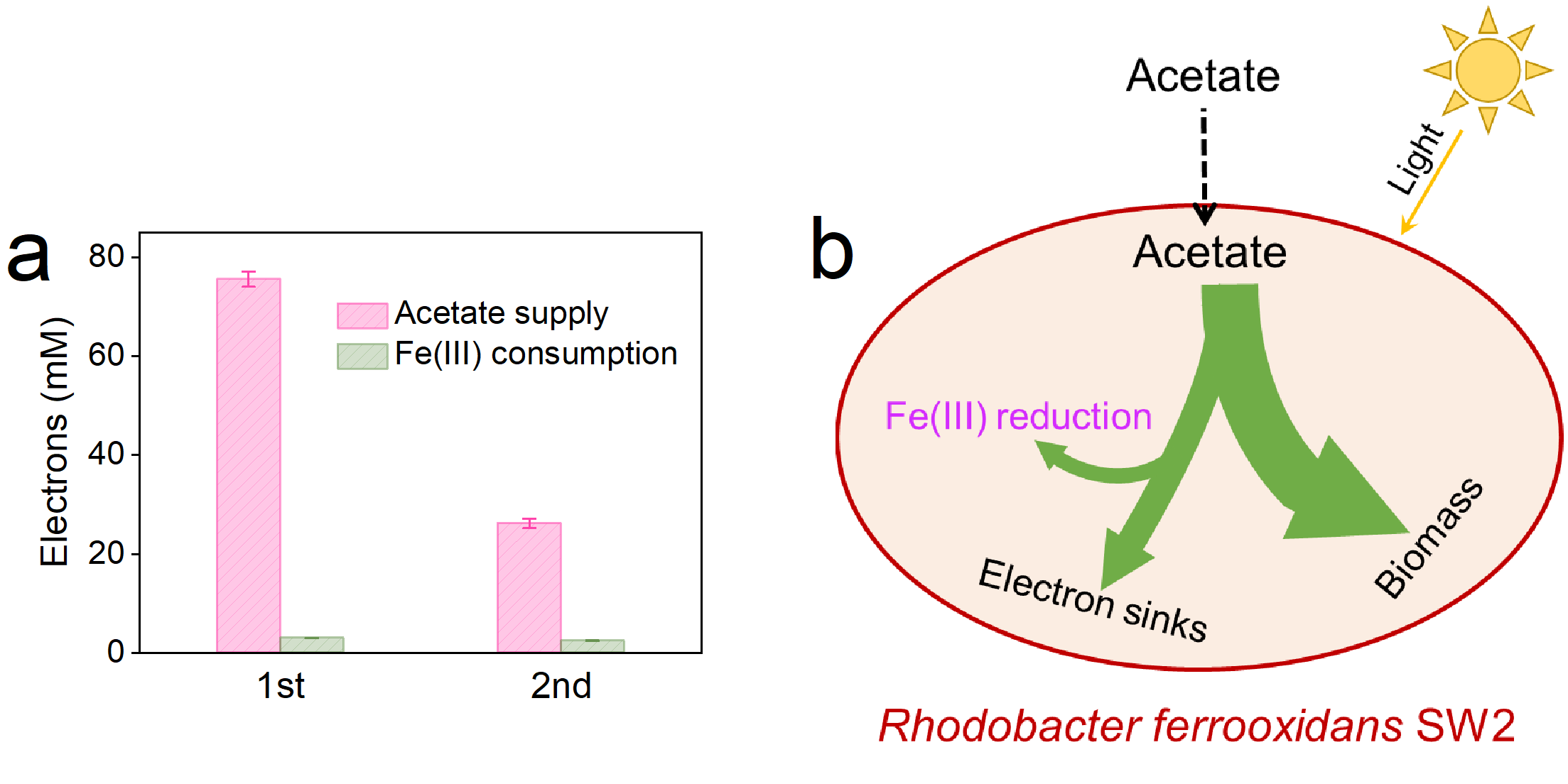

Supplement: Supplementary 1 — Figs. S1 to S10 Tables S1 to S6 [file research.0528.f1.zip › Fig. S1.tif]

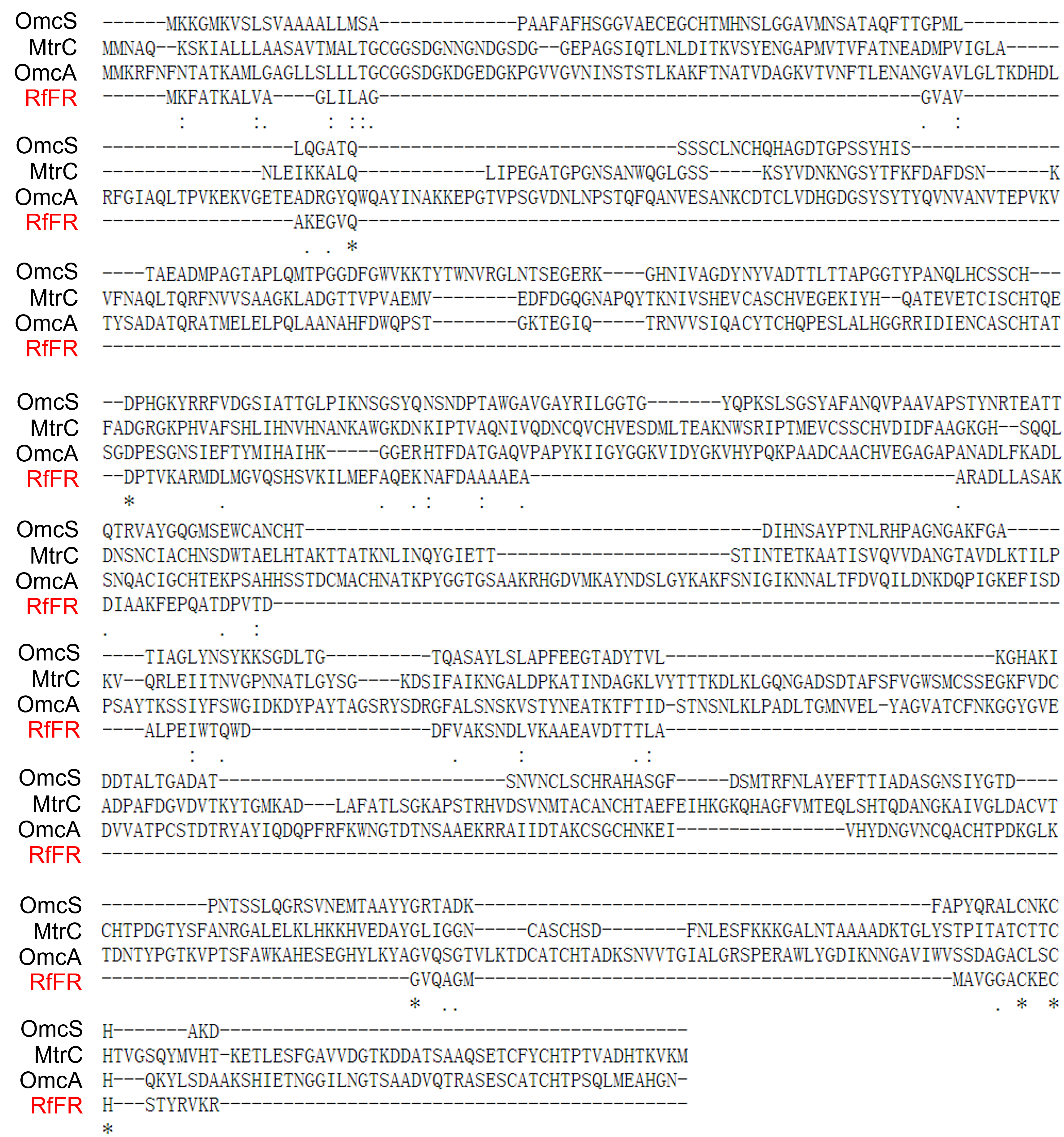

Supplement: Supplementary 1 — Figs. S1 to S10 Tables S1 to S6 [file research.0528.f1.zip › Fig. S10.tif]

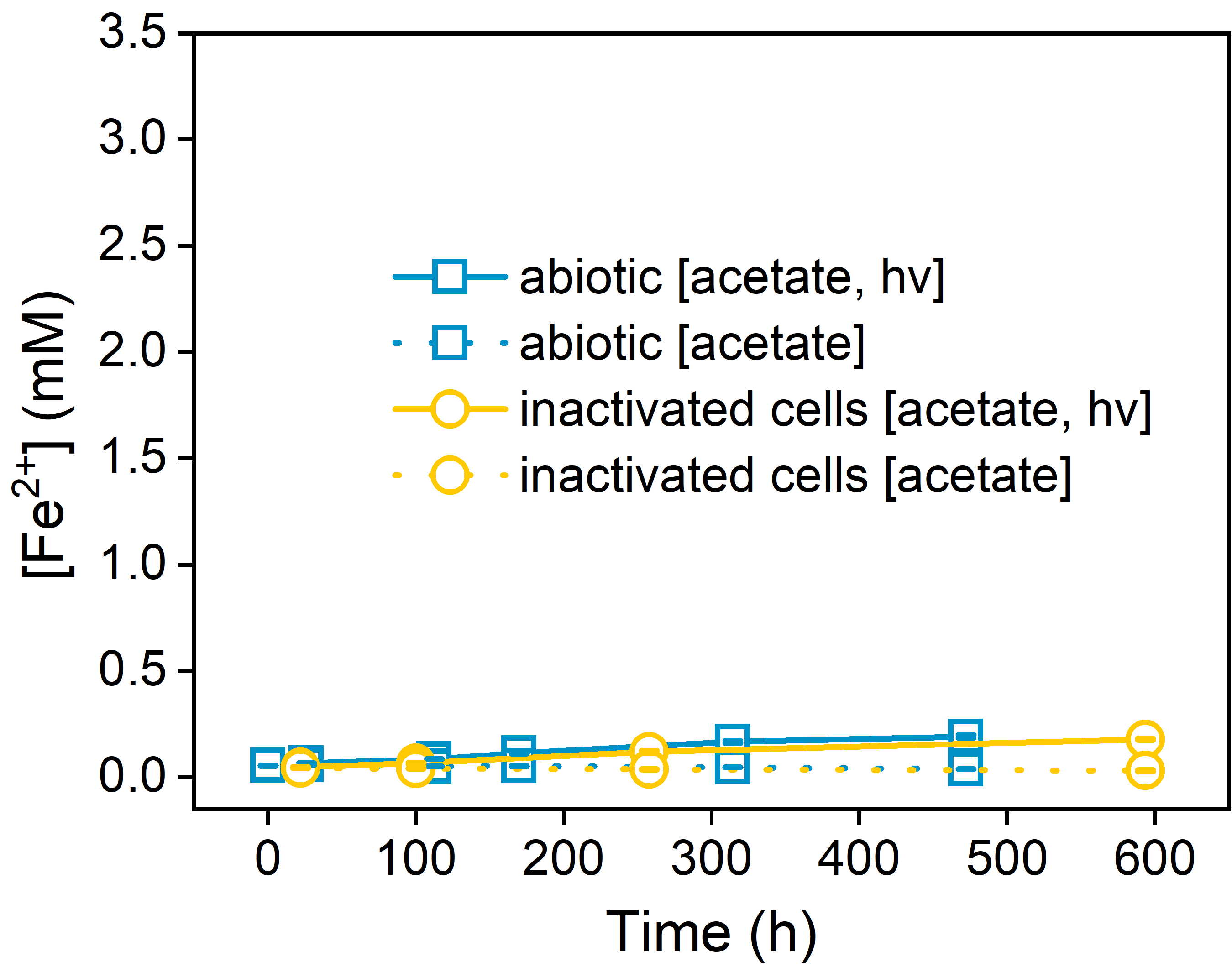

Supplement: Supplementary 1 — Figs. S1 to S10 Tables S1 to S6 [file research.0528.f1.zip › Fig. S2.tif]

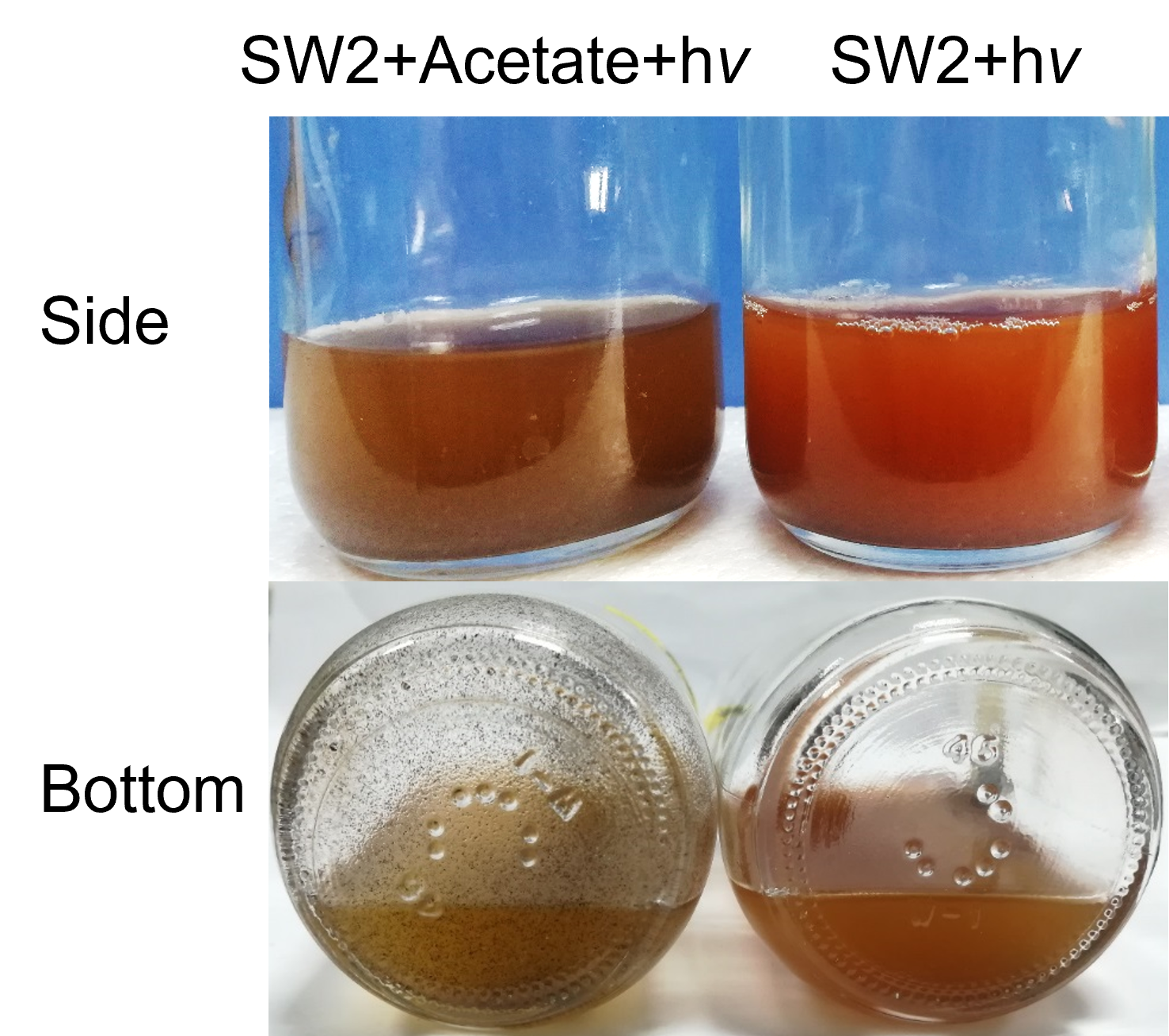

Supplement: Supplementary 1 — Figs. S1 to S10 Tables S1 to S6 [file research.0528.f1.zip › Fig. S3.tif]

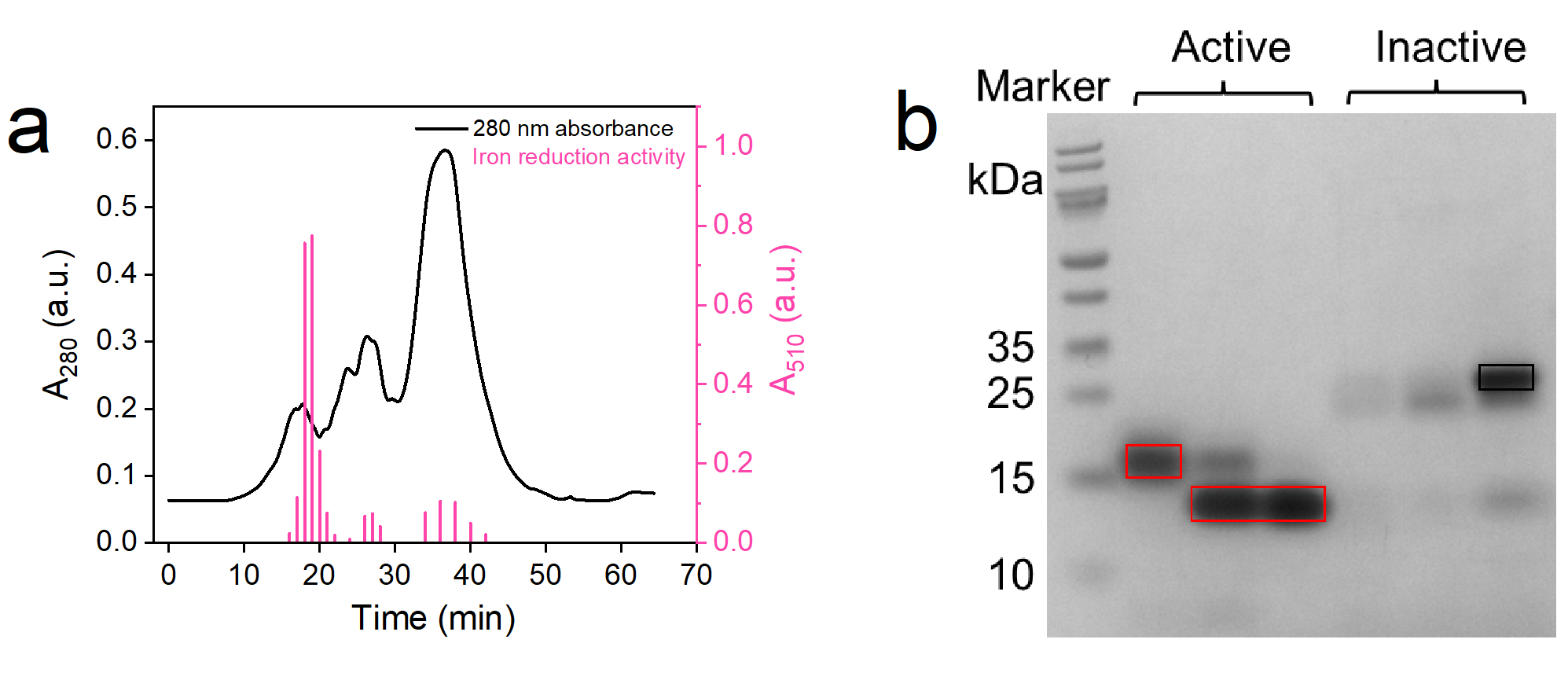

Supplement: Supplementary 1 — Figs. S1 to S10 Tables S1 to S6 [file research.0528.f1.zip › Fig. S4.tif]

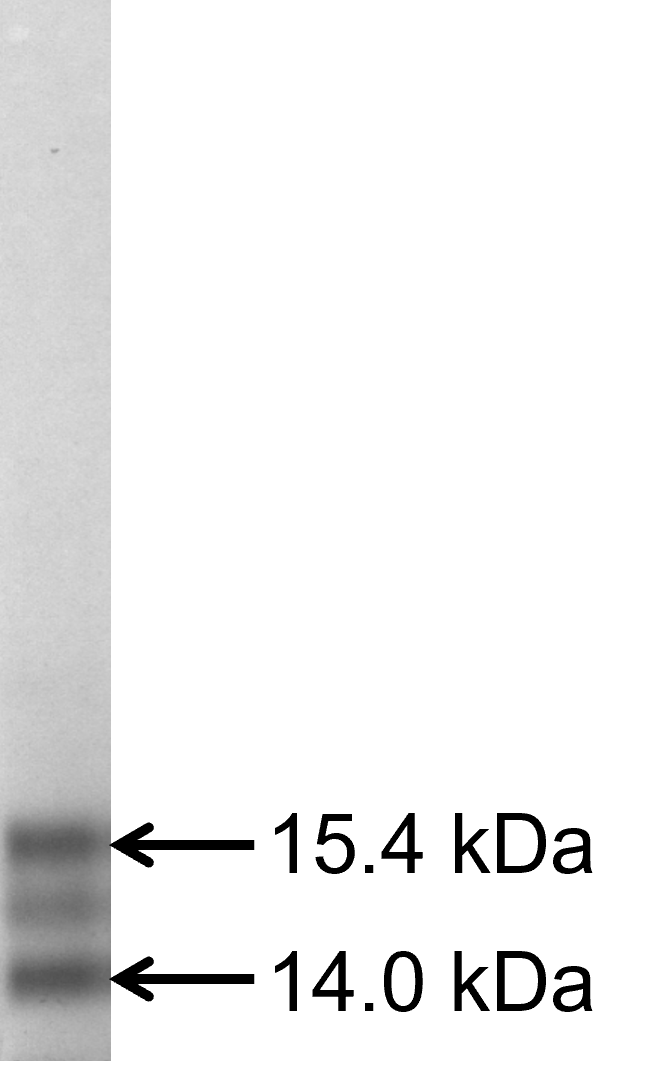

Supplement: Supplementary 1 — Figs. S1 to S10 Tables S1 to S6 [file research.0528.f1.zip › Fig. S5.tif]

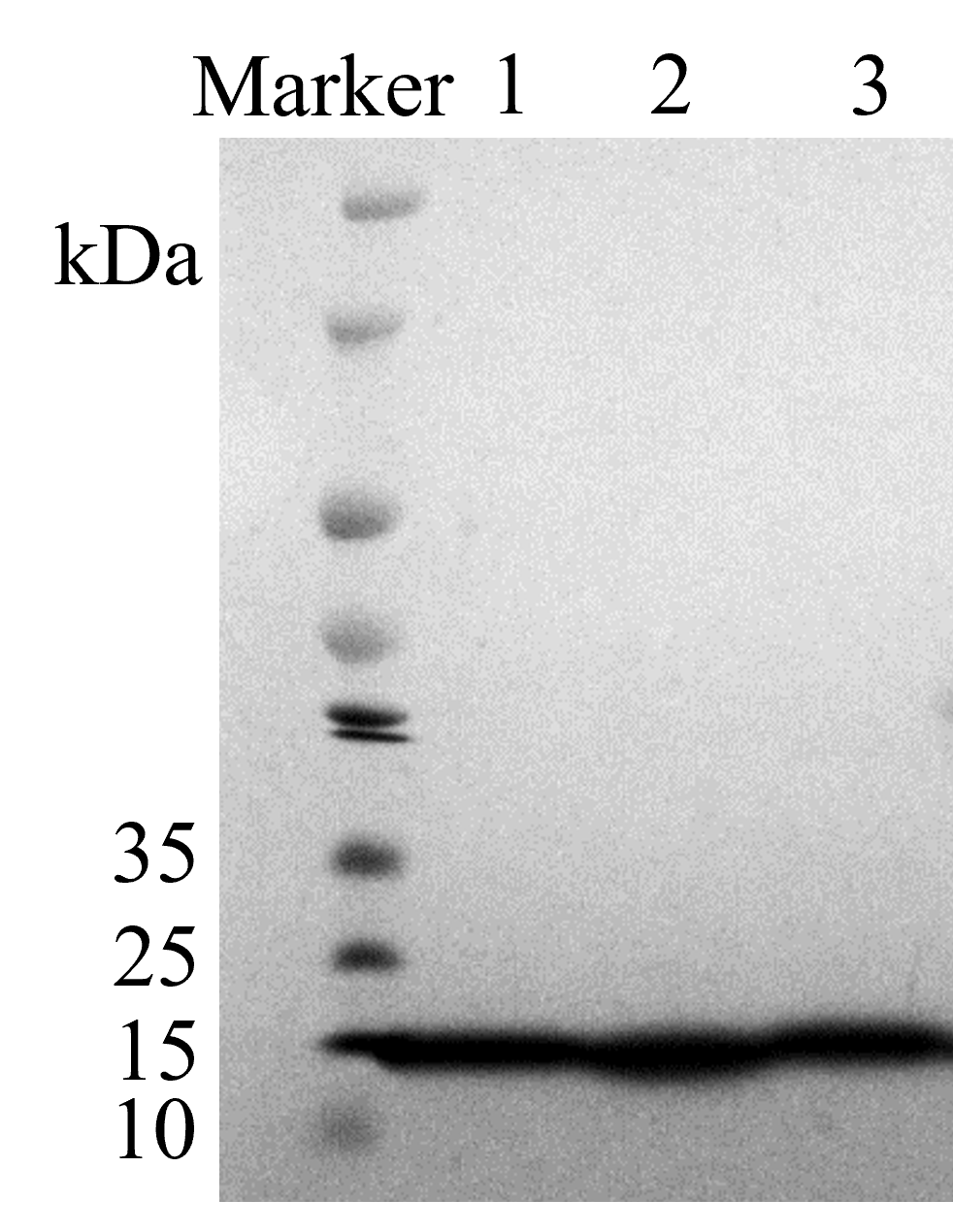

Supplement: Supplementary 1 — Figs. S1 to S10 Tables S1 to S6 [file research.0528.f1.zip › Fig. S6.tif]

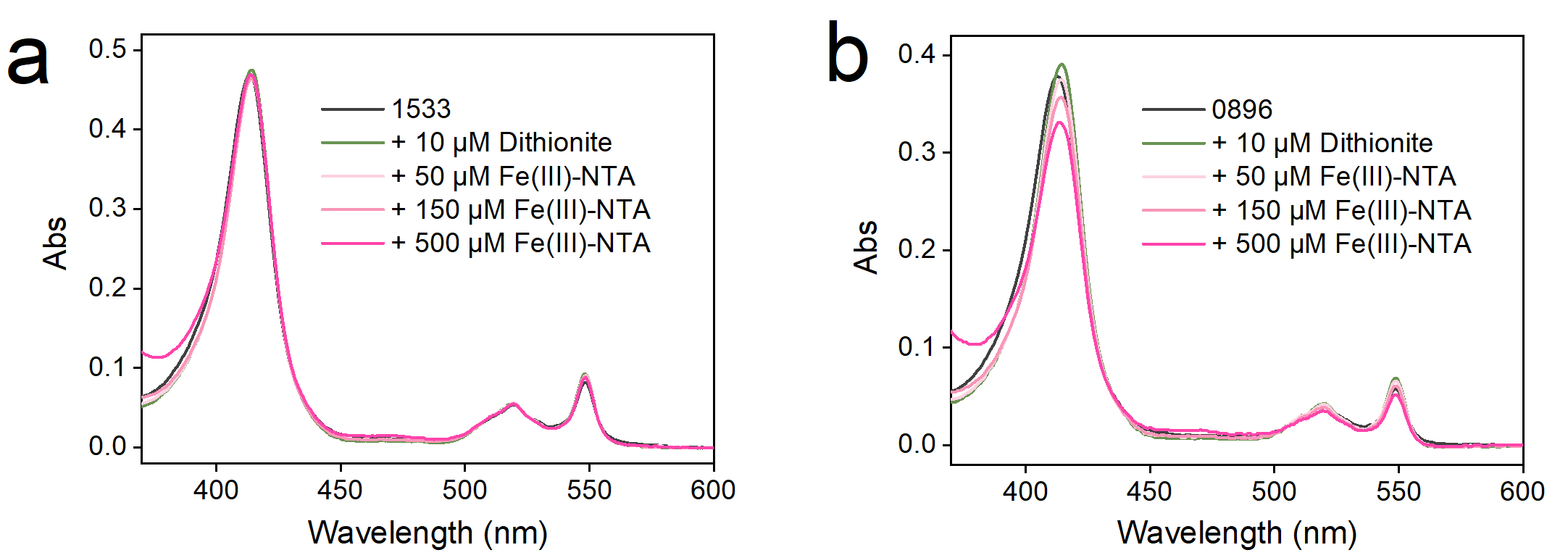

Supplement: Supplementary 1 — Figs. S1 to S10 Tables S1 to S6 [file research.0528.f1.zip › Fig. S7.tif]

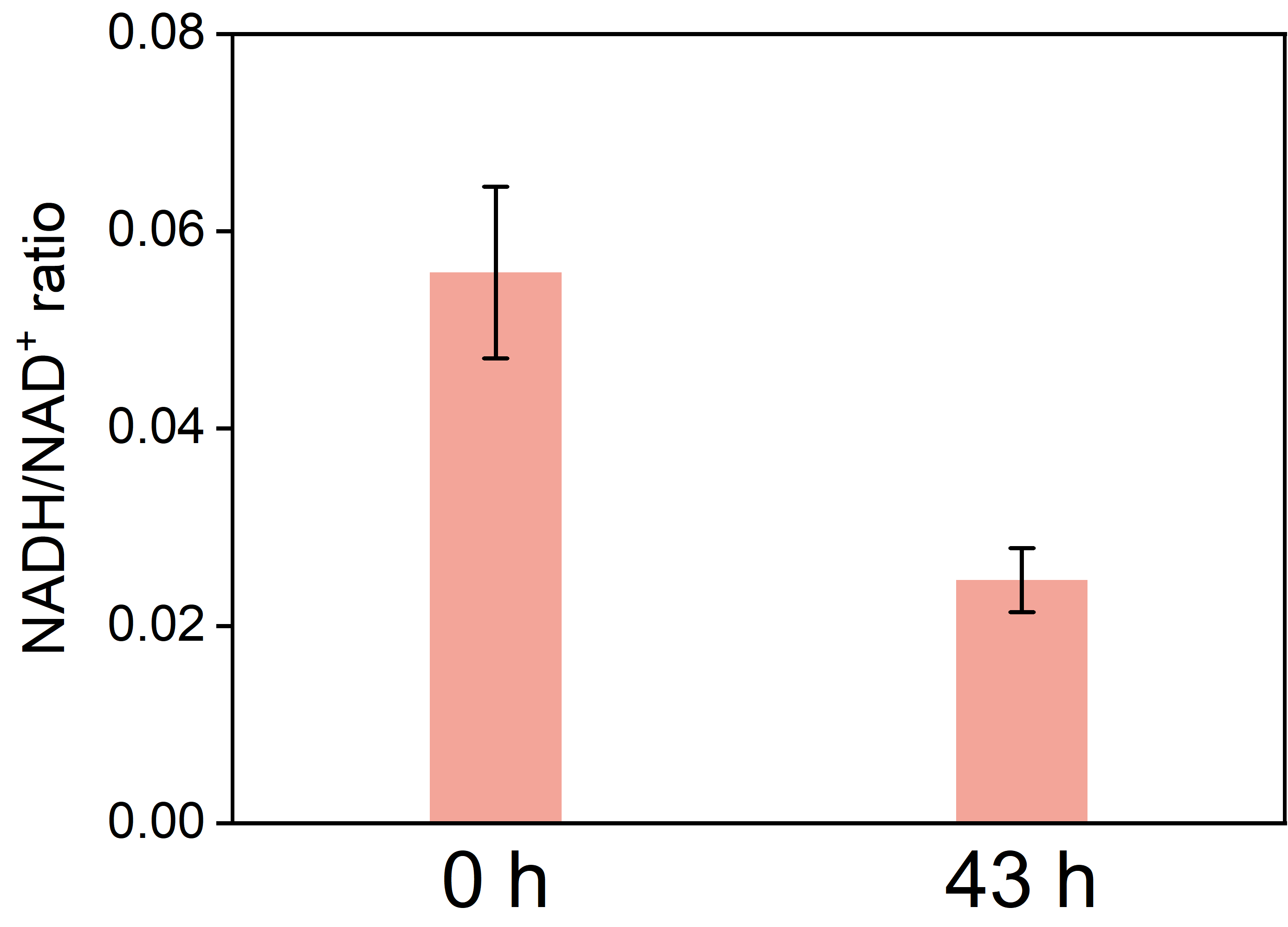

Supplement: Supplementary 1 — Figs. S1 to S10 Tables S1 to S6 [file research.0528.f1.zip › Fig. S8.tif]

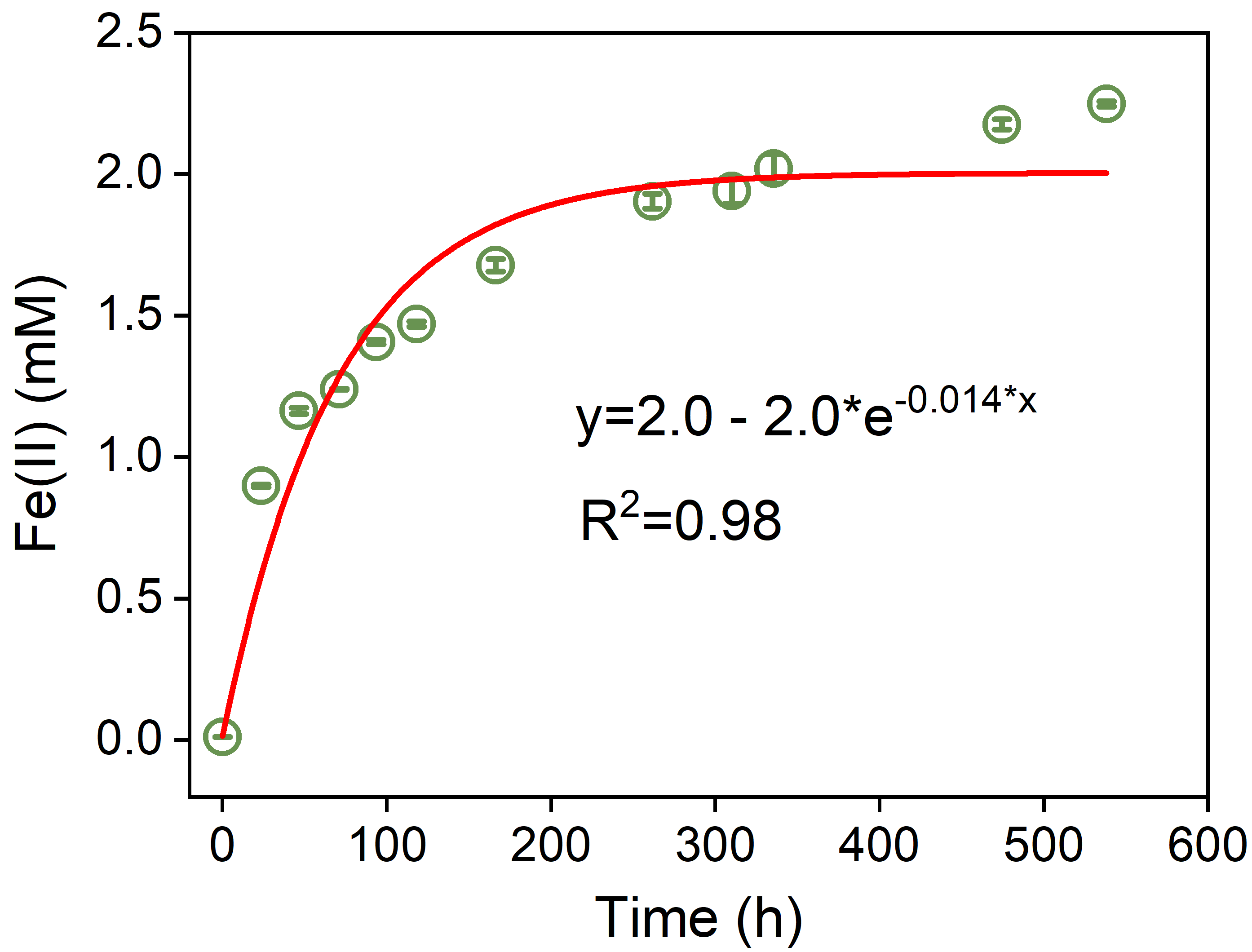

Supplement: Supplementary 1 — Figs. S1 to S10 Tables S1 to S6 [file research.0528.f1.zip › Fig. S9.tif]
